# Targeting cancer stem cells with CAR-based immunotherapy: biology, evidence, and future directions

**DOI:** 10.1186/s12935-025-03846-3

**Published:** 2025-07-28

**Authors:** Kaveh Hadiloo, Parsa Mostanadi, Ali Asadzadeh, Siavash Taremi, Abdolreza Esmaeilzadeh

**Affiliations:** 1 Department of Surgery, Velayat Clinical Research Development Unit, Qazvin University of Medical Sciences, Qazvin, Iran; 2 Student Research Committee, Department of Immunology, Zanjan University of Medical Science, Zanjan, Iran; 3 School of Medicine, Zanjan University of Medical Sciences, Zanjan, Iran; 4 Profound Future Focused Innovative Cell and Gene Therapy, Pficell R&D Canadian Institution & Corporation, Ontario, Canada; 5 Cancer Gene Therapy Research Center (CGRC), Zanjan University of Medical Sciences, Zanjan, Iran

**Keywords:** Chimeric antigen receptor, Cancer stem cells, Tumor initiation, Targeted therapy, Cell-based therapy, Immunotherapy

## Abstract

Cancer stem cells (CSCs) are pivotal in tumor initiation, progression, and relapse, underscoring the need for targeted therapies to achieve lasting responses. This review delves into CSC biology, highlighting their tumor-initiating potential demonstrated through limiting dilution assays and their role in resistance to therapies. Although successful CAR therapies, such as anti-CD19 CAR T-cells, can induce complete responses without directly targeting CSCs, CAR strategies focusing on CSCs may offer promising avenues to prevent recurrence. We assess CAR therapies targeting CSC-specific antigens, including CD133 and GD2, in preclinical and clinical contexts, emphasizing their effectiveness against glioblastoma, breast cancer, and other malignancies. Nevertheless, challenges such as marker specificity and suppression by the tumor microenvironment (TME) persist. Future strategies, which may include dual-targeting and AI-driven marker discovery, aim to improve CSC elimination and advance personalized cancer immunotherapy.

## Introduction

Cancer has an intelligent structure and is utterly adaptive to various situations and treatments. Many studies worked on cancer treatment, but this disease resisted the therapy and survived. In recent years, the more knowledge about immune system construction and the various duties of the compartments, the more novel approaches were introduced, and subsequently, new drugs have been approved. The chimeric antigen receptor (CAR)-based approaches are one of the critical novel anti-tumor therapies introduced by the Eshhars group, which has shown significant efficacy, particularly in hematological malignancies [1]. These receptors enhance the capabilities of various immune cells, with T-cells being the primary focus. CAR T-cell therapy has progressed to clinical application through numerous in vitro and in vivo studies. The Food and Drug Administration (FDA) has approved six CAR T-cell therapies for clinical use. They consist of four anti-CD-19 CAR T cells, including Tisagenlecleucel, Axicabtagene Ciloleucel, Lisocabtagene maraleucel, and Brexucabtagene Autoleucel, as well as two anti-BCMA CAR T cells named Idecabtagene vicleucel and Ciltacabtagene autoleucel [2]. Additionally, two CAR T-cell therapies from Asia have received approval for use in their respective countries. Beyond T-cells, other innate immune system components, such as natural killer (NK) cells, natural killer T (NKT) cells, and macrophages, have been engineered with CARs, showing encouraging outcomes—sometimes surpassing T-cell efficacy in certain studies [2].

Conversely, CAR-based immune cells can target the various antigens and recognize and eliminate tumor cells. One of the best ones is cancer stem cell (CSC)-based antigens. The CSCs’ various features distinguish them as great candidates from other tumor antigens. For instance, tumorigenesis due to robust self-renewal, metastasis, expansion heterogeneity, particular signaling pathways, drug resistance, high expression of anti-apoptotic protein, and restoring the destructed DNA [3]. All factors mentioned could be reasons for resistance/recurrence (r/r) treatment. So, combining CAR-based therapy and CSCs enables a unique treatment opportunity for various cancer types. In recent years, CSC-targeting CAR armored cells have had many experiences in vitro and in vivo, while clinical trials are limited. Despite the low experiences in this manner, the first outcomes demonstrated promising results versus tumors. In this regard, we explain the various aspects of anti-CSC CAR therapy, discuss their achievement in multiple cancers, and discuss the challenges and solutions in this manner.

## Cancer stem cells: the structure, roles, and opportunities in targeting them for cancer treatment

**Fig. 1:**
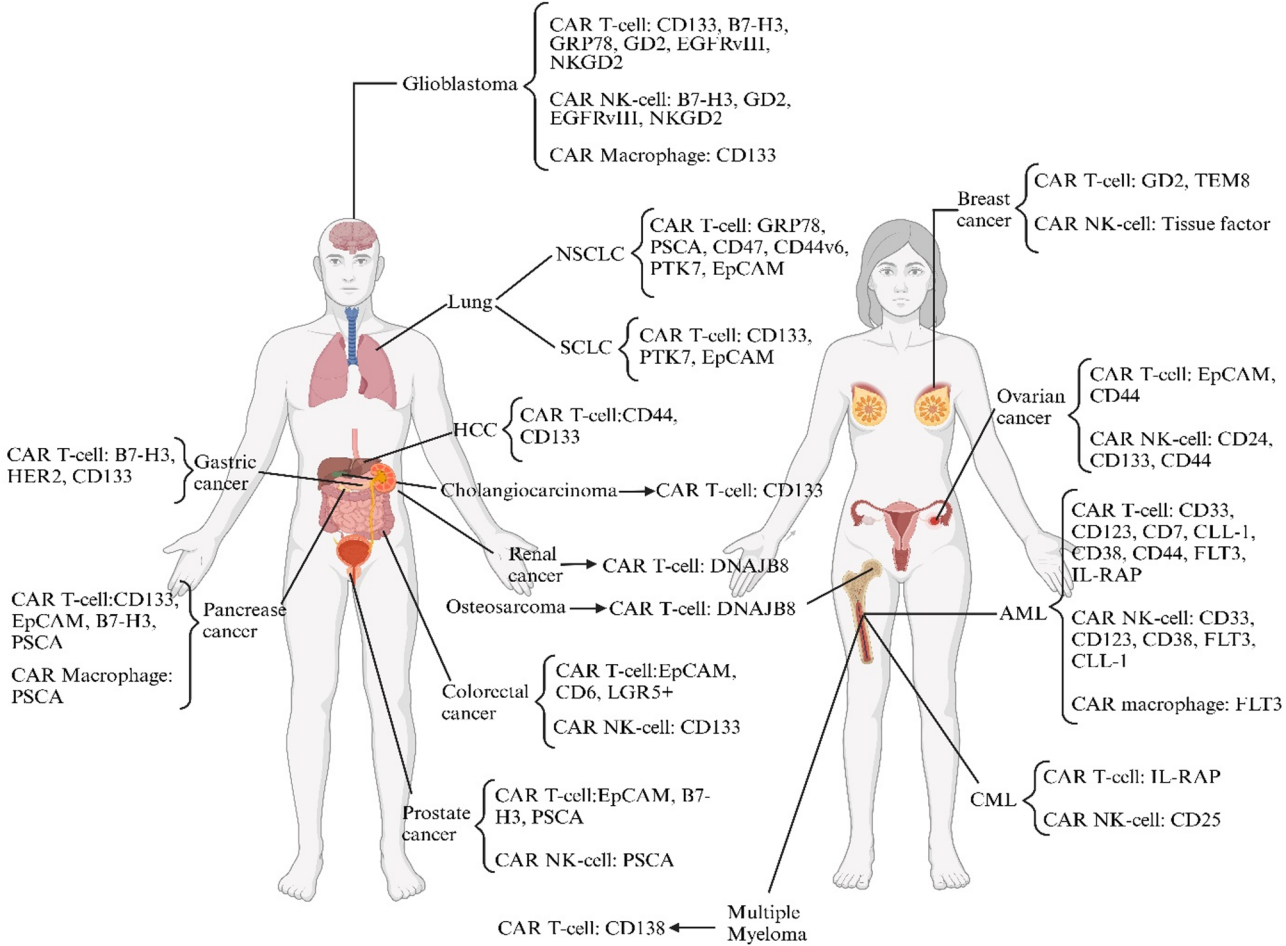
The various CSCs are targeted by CAR-armored cells. As shown above, CAR-armored cells could target the different tumor CSCs in preclinical and clinical settings

CSCs represent a small yet pivotal subset of tumor cells that orchestrate tumor initiation, progression, and resistance to conventional therapies (Fig. 1). Defined by their capacity for self-renewal, differentiation into heterogeneous progeny, and tumor reconstitution, CSCs are a powerful driver of malignancy [4]. These cells exhibit distinct biological features compared to non-CSCs, including reliance on oxidative phosphorylation or aerobic glycolysis for energy, asymmetric division to maintain stemness, and heightened expression of anti-apoptotic proteins and DNA repair mechanisms [5]. Such traits enable CSCs to survive chemotherapy, radiotherapy, and immune surveillance, contributing to tumor relapse and recurrence [6].

Additionally, limited dilution transplant assays highlight the potency of CSCs: in glioblastoma, just 100 CD133 + cells are capable of initiating tumors in mice, whereas 100,000 CD133- cells do not produce tumors [7]. These assays quantify CSC frequency and validate their hierarchical role over clonal evolution models, which attribute growth to random mutations [3].

The origins of CSCs remain a subject of intense investigation. One prevailing theory suggests that CSCs arise from normal stem cells that acquire oncogenic mutations, endowing them with uncontrolled proliferative potential [8]. Alternatively, non-CSCs may dedifferentiate into CSC-like states through epigenetic reprogramming or microenvironmental cues, highlighting their plasticity [9]. This plasticity is regulated by key signaling pathways—Notch, Hedgehog, Wnt/β-catenin, JAK/STAT, and NFκB—which sustain CSC maintenance and vary across cancer types [4]. For instance, Wnt/β-catenin drives colorectal CSC survival, while Notch predominates in glioblastoma stem cells (GSCs) [10]. CSC heterogeneity further complicates their biology, as subpopulations within a tumor may exhibit distinct molecular profiles, metabolic adaptations, and therapy resistance mechanisms, challenging uniform therapeutic targeting [11].

Two models explain tumor progression and diversity: the clonal evolution and CSC models. The clonal evolution model posits that random mutations confer selective advantages to specific cell populations, driving tumor growth. In contrast, the CSC model asserts that a hierarchical organization led by CSCs capable of asymmetric division sustains tumor complexity [3]. The latter model better accounts for the hierarchical heterogeneity observed in high-grade malignancies, where CSCs are enriched, whereas clonal evolution struggles to explain benign tumor dynamics [8]. Experimental evidence, such as the ability of CD133 + glioblastoma cells to recapitulate tumors in xenografts, supports the CSC model’s relevance [7].

CSCs possess unique features that render them attractive therapeutic targets. These include elevated intracellular enzyme activity (e.g., aldehyde dehydrogenase (ALDH)), mitochondrial membrane potential, reactive oxygen species (ROS) scavenging, and autofluorescence [5]. Their resistance to cytotoxic therapies stems from upregulated drug efflux pumps (e.g., ABC transporters), reduced ferroptosis, and enhanced autophagic activity [6]. However, conventional treatments like chemotherapy and radiotherapy often fail to eradicate CSCs, sometimes promoting CSC-like traits through senescence induction or TME-mediated plasticity [11]. For example, chemotherapy-induced senescence can arrest tumor cells in G0 while increasing stemness markers like CD44, fostering recurrence [3]. Thus, targeting CSC-specific surface antigens or signaling pathways offers a promising strategy to disrupt tumor hierarchies.

While CSC characteristics are broadly conserved, their markers and driver pathways vary by cancer type (Fig. 1). In high-grade tumors, CSCs are more prevalent due to increased heterogeneity, mutation burden, and dysregulated division, making them critical targets for aggressive cancers [10]. Eliminating CSCs can induce complete responses. Preclinical studies show anti-CD133 antibodies in colorectal cancer reduce tumor burden and prevent regrowth, unlike bulk-targeting therapies [12]. In AML, targeting CD44 + LSCs with monoclonal antibodies achieves durable remission in xenografts, while sparing CSCs leads to relapse [13]. Conventional therapies often fail—chemotherapy-induced senescence can even enhance CSC traits (e.g., CD44 expression), underscoring the need for CSC-specific approaches [11].

Methods to target CSCs include monoclonal antibodies, pathway inhibitors, metabolic modulators, and immunotherapies like CAR-based approaches [14]. By combining CSC biology with CAR technology, novel therapies can address the root causes of tumor persistence, offering hope for improved clinical outcomes.

## Chimeric antigen receptor-based therapy revolutionized the cancer treatment

The CAR structure has several domains vital for signaling and activation against tumor cells, with different immune cells having unique activation pathways. It comprises three main parts: intracellular, extracellular, and transmembrane domains. The extracellular domain includes a single-chain variable fragment (ScFv), which enables CAR-engineered cells to recognize specific antigens on target T-cells [15]. The intracellular domain of the CAR is crucial for initiating anti-tumor signaling. CAR structures are categorized into five generations, with the first generation lacking a co-stimulatory domain. Subsequent generations have variations in co-stimulatory domains, enhancing the immune cells’ capabilities [16].

CAR types provide another way to categorize cancer treatments. Cancer resistance to various therapies and patient responses influence CAR structure. Each type has specific antitumor capabilities and aims to reduce the side effects of treatment. New CAR types are constantly being developed, including converter, SUPRA, inducible, physiological, dual, switch, and synNotch CARs, each with unique tumor elimination features [17]. Based on the various categorized CAR generations and types, their use must be situation consideration treatment process and patient situation. Indeed, the other immune cells utilized in the CAR showed beneficial outcomes in the preclinical and clinical settings. Due to the special features, the innate immune system cells like NK, NKT, and macrophage cells could achieve better results than T-cells in some settings, so they may be used further in future studies that our team reviewed in previous articles [18–20]. By the way, the CAR T-cell, as a pioneer of CAR-based therapy, had more investigations and achievements but must wait for more surveys with other immune cells. As a result, the CAR-targeted CSCs are promising candidates for tumor targeting and elimination.

**Table 1:** Comparison of CAR generations and CAR types

Feature	Description	Key aspects
CAR Generations	The CAR structure includes extracellular, transmembrane, and intracellular domains, and it is classified into five generations based on intracellular co-stimulatory domains.	Extracellular: ScFv for antigen recognition. Intracellular: Signaling for anti-tumor response. 1st generation: No co-stimulatory domains. Later generations: Enhanced activation with co-stimulatory domains.
CAR Types	Variations are designed for specific therapeutic goals and to overcome resistance.	Novel CAR types, such as Converter, SUPRA, inducible, physiological, dual, switch, and synNotch, have unique tumor elimination features and aim to reduce side effects.

Abbreviations: CAR: Chimeric antigen receptor, ScFv: Single-chain variable fragment

## CAR targeting CSC, a novel milestone in the cell-based treatment

**Fig. 2:**
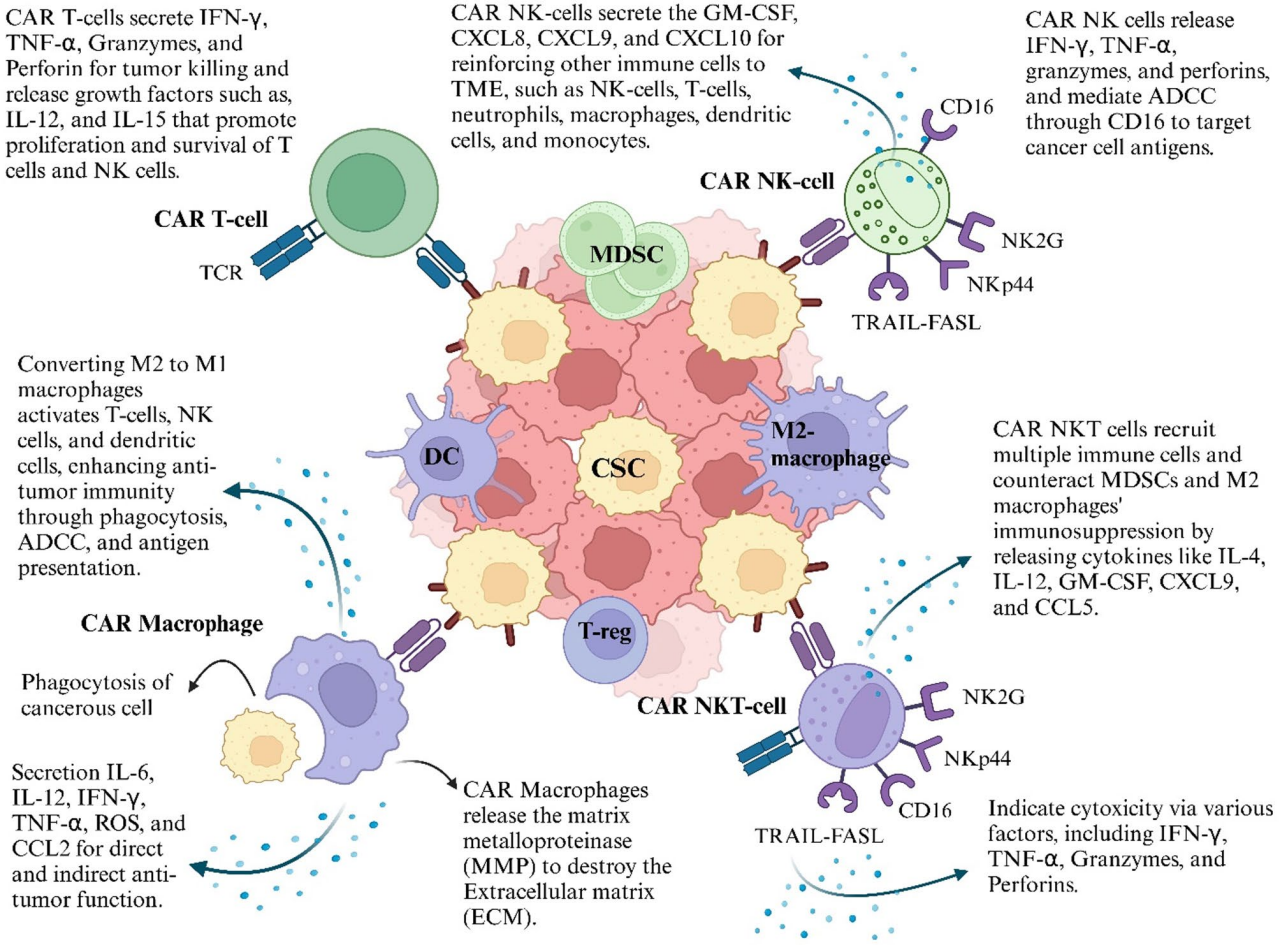
The various CAR-armored immune cells. The CAR structure can be acquired in the innate immune system platform. Providing each immune cell with a CAR structure gives it a distinct capacity to target and eliminate tumors. Abbreviation: MDSC: myeloid-derived suppressor cell, DC: dendritic cell, T-reg: T-regulatory cell, ADCC: antibody-dependent cellular toxicity, GM-CSF: Granulocyte-macrophage colony-stimulating factor, ROS: Reactive oxygen species, CSC: Cancer stem cell

Given the aggressive nature of CSCs, characterized by self-renewal, resistance to apoptosis, and evasion of chemo/radiotherapy and immune defenses, innovative therapeutic strategies are essential to address one of cancer treatment’s most significant challenges. CAR-based therapies are particularly promising for targeting CSCs, as they focus on specific CSC antigens, offering a more effective treatment option with positive outcomes observed in early clinical studies. Multiple CAR-based therapies have recently been applied to target CSCs, employing various immune cells, including CAR T-cells, CAR NK cells, and CAR macrophages (CAR M) (Fig. 2). However, CAR NKT-cells, which show potential in this context, have yet to be utilized.

The efficacy of CAR-based therapies hinges on selecting appropriate tumor antigens, with CSC markers being prime candidates due to their role in tumor maintenance [4]. However, CSC markers range from highly specific (e.g., CD133) to broadly expressed TAAs with CSC relevance (e.g., CD123) [7, 21]. To clarify their context, this review categorizes markers as “CSC-enriched” (predominantly expressed on CSCs), “CSC-associated” (enriched in CSC subsets with functional significance), or “TAAs with CSC relevance” (expressed on both CSCs and bulk tumor cells). This distinction reflects the practical focus of CAR studies on targeting resistant, stem-like populations rather than absolute CSC exclusivity.

Identifying and screening CSC-specific antigens is critical for developing effective CAR-based immunotherapies. Various approaches are employed to identify and validate antigens uniquely or preferentially expressed on CSCs. Gene expression profiling, such as RNA sequencing (RNA-seq) and microarray analyses, helps identify upregulated genes in CSC populations compared to non-CSCs [22]. Proteomic approaches, including mass spectrometry and flow cytometry, enable high-throughput screening of surface markers specific to CSCs. Also, single-cell sequencing is an emerging technique that identifies heterogeneity within CSC populations, helping to pinpoint unique markers [23, 24].

Screening strategies are essential for validating these antigens as therapeutic targets. In vitro validation using CSC-enriched cultures helps assess antigen expression and functional relevance [25]. Animal models, such as patient-derived xenografts, provide in vivo evidence of CSC targeting by CAR T cells. By integrating these approaches, researchers can enhance the specificity and efficacy of anti-CSCs CAR T cell therapies [26].

The reasoning behind using CAR-based therapies to target these elusive cancers requires a comprehensive explanation. CAR T-cell therapy was the first CAR-based treatment approved and has been utilized to combat CSCs in various cancers, including acute myeloid leukemia (AML) and breast cancer [27, 28]. The CAR T-cells can eliminate tumor cells without needing major histocompatibility complex-I (MHC-I) presentation, making them highly suitable for targeting CSCs that may downregulate MHC-I expression [15]. It is essential to differentiate between CSCs and non-cancer stem cells to avoid toxicity to healthy cells. CAR T-cells can target tumors while preserving normal tissue. However, the on-target, off-tumor effect is a significant challenge in using CAR T-cells to target CSCs. This issue occurs because specific CSC markers, like CD133 and ALDH, are also present in normal cells [29, 30]. While CAR T-cell therapy has significantly advanced cancer treatment, it comes with several drawbacks, such as restricted tumor infiltration in solid tumors, cytokine release syndrome (CRS), neurotoxicity, graft-versus-host disease (GvHD), and the need to produce CAR T-cells from autologous T-cells [31]. These challenges emphasize the importance of developing alternative CAR-based therapies that harness the power of the innate immune system, such as CAR-NK cells and CAR-macrophages.

CAR NK cells offer several advantages over CAR T-cells. For instance, unlike T-cells, NK cells do not require MHC I recognition, meaning they can be activated without autologous NK cells. This feature allows CAR NK cells to be used in off-the-shelf applications [31, 32]. Furthermore, CAR NK cells are associated with lower levels of CRS and neurotoxicity than CAR T-cells, as they produce granulocyte-macrophage colony-stimulating factor (GM-CSF) instead of IL-2, IL-6, and TNF-α. Additionally, CAR NK cells can eliminate tumors through CAR-dependent and CAR-independent mechanisms, unlike CAR T-cells and CAR-M cells [31]. This capability may allow CAR NK cells to eliminate CSCs based on their functional properties and lack of surface antigens rather than relying on the presence of positive antigens on the CSCs. This innate cytotoxicity makes CAR NK cells a versatile option for targeting diverse types of cancer cells. Although CAR NK cells overcome several problems associated with CAR T-cells, short half-life, limited tumor infiltration in solid tumors, and loss of their function under immunosuppressive conditions in TME still needs to be solved [31].

For example, a phase I/II clinical trial (NCT03056339) utilizing CD19 targeting CAR NK cells derived from umbilical cord blood in B-cell malignancies demonstrated an objective response rate of 73% with no CRS or GVHD [33]. Also, preclinical models have highlighted the superior tumor penetration of CAR NK cells compared to CAR T cells, particularly in solid tumors, due to their ability to traffic through stromal barriers more efficiently than CAR T cells. In addition, investigations indicate that engineering CAR NK cells to secrete IL-15 enhances their persistence and survival, addressing the short half-life observed in standard NK cell therapies [34–36].

Immunosuppression in the TME and challenges in cell trafficking for treating CSCs pose significant hurdles that require innovative immunotherapy approaches. Due to the obstacles that CAR T-cells and CAR NK cells face in overcoming these challenges, employing CAR M against CSCs offers promising advantages. Firstly, macrophages can infiltrate solid tumors, overcoming cell trafficking issues [18]. The hypoxic conditions in the TME induce the secretion of cytokines, including CCL2, CXCL12, CSF1, and vascular endothelial growth factor A (VEGF-A), which provoke macrophages to infiltrate tumors and become retained within them [37]. Another advantage of CAR M is its resistance to the immunosuppressive environment of the TME, an essential factor in the effective treatment of CSCs due to high immunosuppressive cells around the tumors [18, 31]. Tumor-associated macrophages (TAMs), the main population of immune cells in the TME, typically exhibit M2-like and pro-tumoral signatures and can be converted to M1 macrophages by CAR M. This conversion enhances cytotoxicity in the TME while ensuring that TAMs do not impair the function of CAR M [18]. Although CAR M has overcome several barriers, some limitations necessitate further study. Investigating in vivo dosing requirements, side effects, cost, and tumor recurrence is necessary. Additionally, their safety and efficacy in humans need to be ensured. Another barrier is that, unlike T-cells, macrophages cannot spread throughout the body, which presents a significant problem in the distribution of CAR Ms [18].

Another promising CAR-based therapy for targeting CSCs is CAR NKT-cells, which may offer a viable alternative to both CAR NK cells and CAR T-cells due to several advantages. For example, CAR NKT-cells exhibit better safety profiles compared to CAR T-cells, with reduced side effects, a lack of GvHD response, improved tumor infiltration, and the ability to stimulate dendritic cells (DCs) and CD8 + T-cells for long-term immune responses. Additionally, CAR NKT cells offer enhanced immune regulation and increased persistence compared to CAR NK cells. However, like other CAR-based therapies, CAR NKT-cells face limitations, including a limited supply of NKT-cells from the source, decreased cytotoxic activity in the TME, low persistence in the body, and a complex manufacturing process [19].

Consequently, current CAR therapies have limitations in targeting CSCs, but each approach offers advantages and disadvantages that may be better suited for specific CSC types or tumor microenvironments.

## The potential markers of CSCs targeted by CAR-based treatment

Many CSC antigens have been targeted using the CAR structure and accompanied by various results (Table 1). Some of these data benefit the CAR effects in targeting CSCs, but the outcome is converse in some cases. By the way, the CAR-targeted CSC efficacy of each cancer type must be evaluated. While some CAR-based therapies have achieved durable responses in hematological cancers without targeting CSCs, CSC-focused CAR therapies effectively address relapse by eliminating tumor-initiating cells. Preclinical and clinical data demonstrate their potential across various cancer types.

### Hematological cancer

#### Acute myeloid leukemia (AML)

Several TAAs, such as CD123, CD33, CD7, C-type lectin-like molecule 1 (CLL-1), CD34, CD38, CD44, CD90, CD135, CD371, FMS-like tyrosine kinase 3 (FLT3), Lewis Y (LeY), interleukin-1 receptor accessory protein (IL-1RAP), and ALDH, have been identified as markers for CSCs [14, 38].

In preclinical and clinical studies, CD33, CD123, CLL-1, and CD7 are the most frequently targeted by CAR-based therapies, including CAR T-cells and CAR NK cells. Additionally, a study has explored CAR M targeting FLT3 in preclinical trials.

CD33 is enriched in AML LSC fractions, justifying their use in CAR trials despite the broader expression. Several dual-targeting CAR T-cell strategies have been developed to enhance specificity and minimize on-target, off-tumor toxicity. One such approach involved using dual-CAR cytokine-induced killer (CIK) cells, combining a low-affinity anti-CD123 IL-3zetakine with an anti-CD33 costimulatory receptor. This strategy targeted CD123+/CD33 + AML cells while reducing damage to healthy cells [39]. Another dual-targeting strategy involved engineering CD33-CLL1 CAR T-cells, which demonstrated potent cytotoxicity against AML cells while showing limited toxicity toward normal cells, confirming safety and effectiveness in animal models [40]. Additionally, anti-IL10R CAR T-cells secreting CD33-targeting bispecific antibodies (BsAbs) were employed to improve activation and persistence against AML cells with diverse CD33 and IL10R expression levels. This approach enhanced CAR T-cell cytotoxicity while recruiting bystander T-cells for increased tumor targeting, addressing challenges such as poor persistence and antigen loss [41]. Another dual-targeting approach involved the use of TIM3 and CD33 to enhance specificity. TIM3, expressed on LSCs but not healthy hematopoietic precursors, provides a promising target. A study compared various dual-CAR T-cell designs—compound, split, tandem, and pooled approaches—and found that split CAR T-cells targeting TIM3 and CD33 were the most precise, effectively targeting AML cells with both markers while avoiding healthy cells, indicating their potential use without stem cell transplants [42].

Another study introduced DARIC33, a CAR system that utilizes rapamycin to control T-cell activation against CD33 + tumors, offering precise management of CRS, transcription, and cytotoxicity. DARIC33 T-cells activated at low rapamycin doses (1 nM) without impairing CD34 + stem cell colony-forming capacity, and a phase I trial (PLAT-08, NCT05105152) is currently underway [43]. Additionally, CAR-TCR T-cells, which integrate both CAR and TCR therapies within the same T-cell, have shown enhanced anti-tumor effects in animal models, especially in cases with low antigen expression. The combination of CAR T-cells and TCR-T-cells proved effective in eliminating AML, whereas using either therapy alone was insufficient for successful treatment [44].

Anti-CD33 CAR T-cells typically recognize the V-set domain of CD33, which is located far from the cell membrane. A study revealed that reducing the distance between T-cells and leukemia cells improves the effectiveness of anti-CD33 CAR T-cells. Second-generation CAR constructs were developed using antibodies targeting the membrane-proximal C2-set domain (CD33PAN antibodies) to address this. This modification enabled CAR T-cells to bind CD33 independently of the V-set domain. CD33PAN-CAR T-cells demonstrated superior tumor clearance, improved survival, and enhanced efficacy in mouse models of human AML, all without causing increased T-cell exhaustion [45].

CAR NK cells present a promising alternative to CAR T-cells, particularly in mismatched HLA settings, and offer a potentially safer approach for treating AML. One study reported that clustered regularly interspaced short palindromic repeats and CRISPR-associated protein 9 (CRISPR-Cas9)-mediated knockout of NKG2A in CD33-CAR NK cells led to enhanced cytotoxicity against AML in vitro, significantly reducing the leukemic burden [46]. The potential of induced pluripotent stem cell (iPSC)-derived NK cells has also been explored. Anti-CD33 CAR iNK cells were engineered with four functional modalities: targeting AML via CD33, enhancing antibody-dependent cell-mediated cytotoxicity (ADCC) through high-affinity CD16, improving cell persistence via IL-15, and preventing NK cell fratricide by knocking out CD38 [47]. In a study, the safety and efficacy of CD-33 CAR NK cells were evaluated in a phase 1 clinical trial. There were no grade 3–4 adverse events (only one patient with grade 2 CRS), and six of 10 patients reached MRD-negative complete remission [48]. These cells demonstrated potent anti-tumor activity against multiple AML cell lines and primary AML blasts.

Overall, these studies underscore the potential of anti-CD33 CAR NK cells as a safer and more effective alternative for AML treatment, with additional genetic modifications further enhancing their therapeutic efficacy. Combining CAR NK technology with gene editing tools, iPSC-derived NK cells, and bifunctional designs represents a significant advancement in developing off-the-shelf therapies for AML and potentially other cancers.

CD123 is a well-documented CSC-associated marker, overexpressed on LSCs and linked to chemotherapy resistance [49]. CAR-based therapies targeting CD123, such as CAR T-cells and CAR NK cells, have yielded diverse results, with several studies exploring dual-targeting strategies to enhance efficacy. Due to M-MDSCs, M2 macrophages, and antigen heterogeneity, single CD123 targeting in AML may not suffice. For instance, one dual-targeted CAR T-cell approach combined CD123 with Natural killer group 2 member D ligands (NKG2DLs), effectively eliminating AML cells and immunosuppressive cells while incorporating an RQR8 marker/suicide gene for safe CAR T-cell elimination via Rituximab [21].

Further studies targeted CD123 and CLL-1, as over 80% of AML cells express both antigens. Dual-targeted CD123/CLL-1 CAR T-cells (123CL CAR T-cells) demonstrated improved antigen coverage and prevention of antigen escape while sparing healthy hematopoietic cells; these cells also include an RQR8 suicide gene for enhanced safety [50]Thus, this combination therapy may be required to evaluate the dual-targeted CD123/CLL-1 CAR T-cells.

Another dual-targeting approach used bispecific CAR T-cells against CD33 and CD123 to reduce on-target off-tumor effects. These bispecific CARs effectively activated T-cells in an “AND” logic manner, minimizing adverse effects while achieving tumor control in an AML mouse model [51].

Certain studies highlight CAR NK cells as safer alternatives for CD123 targeting due to their lower toxicity. Anti-CD123 CAR NK92 cells showed elevated cytotoxic activity and leukemia reduction in vivo [52]. Compared with CD123 CAR T-cells, CD123 CAR NK cells demonstrated significantly lower toxicity in mice, sparing human bone marrow cells, with negligible toxicity in endothelial tissue models, suggesting CAR NK cells could be beneficial in clinical settings for reducing CRS and T-cell exhaustion [53]. Therefore, utilizing CAR NK cells instead of CAR T-cells may lead to better outcomes in clinical trials.

Furthermore, several clinical studies have been conducted on the efficacy of anti-CD123 CAR T-cells, and the outcomes of these studies are reported in Table 1.

CD7 is a promising target for CAR-based therapies because it is expressed in AML cells and T-cells but is absent in myeloid cells, minimizing potential off-target effects. CD7, though less CSC-specific, targets AML subsets with stem-like properties in preclinical models [54]. Among CAR-based therapies, only CAR T-cells have been utilized to target CD7, and in one study, naturally selected CD7 CAR T-cells, which eliminate CD7 expression after transduction and do not require knockdown, effectively eradicated CD7 + AML cells in vitro and in vivo. This approach prevented fratricide by ensuring the CAR T-cells did not recognize their CD7 [54]. Another study showed that pharmacological inhibitors like Ibrutinib and Dasatinib could prevent fratricide in anti-CD7 CAR T-cells in vitro and in vivo, presenting a non-gene-editing alternative [55].

Given the possibility of fratricide occurring by anti-CD7 CAR T-cells, using CAR NK cells to target this antigen without needing pharmacological inhibitors or gene editing may offer a viable alternative to CAR T-cells.

CLL-1 is another notable target in AML therapy, with studies exploring various enhancements to improve CAR T-cell function, such as PD-1 silencing and IL-15 addition [56, 57]The following studies discussed the novel structure of CAR-based therapies for targeting CLL-1 that may be used in clinical models.

To overcome production limitations and reduce costs, a novel allogeneic CAR-T platform called ThisCART was developed based on the intracellular retention of TCR and HLA-I molecules. This platform allows for the efficient production of anti-CLL-1 CAR T-cells. Preclinical results showed that CLL-1 CAR T-cells expanded over 150-fold ex-vivo, with a purity exceeding 99%. The ThisCART-cells demonstrated superior purity, enhanced tumor-killing ability, and successfully avoided GvHD in preclinical studies, highlighting their potential for future clinical applications [58]. Besides CLL-1, other markers such as CD33 and CD123 have also been targeted by dual-targeted CAR T-cells, as discussed previously [40, 50]. Additionally, dual-targeted CD33/CLL-1 CAR T-cells have been evaluated functionally in clinical trials. (Table 1)

In addition, anti-CLL-1 CAR NK cells have been developed using the TC Buster DNA transposon system, allowing clinical-scale expansion from healthy donor cells. These second-generation anti-CLL-1 CAR NK cells demonstrated vigorous anti-AML activity in vitro, with improved persistence achieved through CRISPR/Cas9-mediated CISH gene knockout. This method provides an efficient and scalable option for AML therapy, showing promise for targeting LSCs in clinical settings. The findings suggest that this method is an efficient and scalable platform for producing CAR NK cells for AML therapy, showing significant promise in targeting LSCs [59].

Several studies have investigated CAR-based therapies targeting CD38 in AML. One study developed anti-CD38 CAR T-cells (CART-38), effectively targeting AML, T-ALL, and MM cells in xenograft models and improved survival. While CART-38 did not impact T-cell expansion, it reduced hematopoietic progenitors in humanized models, indicating a need to manage potential toxicity in clinical applications [60]. Another study enhanced CART-38 by downregulating the PI3Kδ pathway with shRNA, reducing cytokine release (e.g., IL-2, IFN-γ, TNF) and improving survival in AML models, highlighting the benefits of reduced side effects alongside vigorous anti-leukemia activity [61].

Despite these advancements, anti-CLL-1 CAR T-cells may be particularly beneficial for relapsed AML cases. In one report, two patients with relapsed/refractory (r/r) AML, who relapsed after transplant and anti-CD38 CAR T therapy, achieved molecular complete remission following treatment with PD-1-silenced anti-CLL-1 CAR T-cells. This demonstrated the potential of anti-CLL-1 CAR T-cells as a safer and more effective treatment option for relapsed AML [62].

Anti-CD38 CAR NK cells have shown significant potential in cancer therapy. Researchers developed fratricide-resistant CD38 CAR NK cells using CRISPR/Cas9 to knockout CD38 while simultaneously inserting the CAR gene, achieving cytotoxicity comparable to CAR T-cells without causing fratricide. Moreover, pre-treating cancer cells with all-trans retinoic acid (ATRA) enhanced CD38 expression, improving NK cell efficacy. This combination of CD38 CAR NK cells with ATRA presents a promising therapeutic strategy for hematologic cancers [63].

CD44 is another potential target for CAR T-cell therapy. A study assessed the efficacy and safety of anti-CD44v6 CAR T-cells in treating AML. CD44v6 expression on T-cells can lead to transient fratricide and exhaustion, reducing CAR T-cell effectiveness, and is linked to DNA methylation. Hypomethylating agents (HMAs), such as decitabine (Dec) and azacitidine (Aza), commonly used in AML treatment, may enhance the effectiveness of anti-CD44v6 CAR T-cell therapy. Treatment with Dec or Aza improved CAR + cell output and induced a memory phenotype, with Dec showing more effectiveness. Both agents triggered apoptosis in AML cells, particularly those with DNMT3A mutations, and increased CD44v6 expression. Combining HMA-treated anti-CD44v6 CAR T-cells with AML cells yielded the most potent anti-tumor effects, suggesting this could be a promising therapeutic strategy for AML [64].

FLT3 is frequently activated in AML through mutations or overexpression in KMT2A-rearranged ALL, both associated with poor outcomes. Researchers developed anti-FLT3 CAR T-cells (FLT3CART) and bispecific CD19xFLT3CART, demonstrating strong cytokine responses and cytotoxicity against FLT3-mutant AML and KMT2A-rearranged ALL. FLT3CART inhibited leukemia proliferation in xenograft models, including a patient-derived lineage-switched model, while CD19xFLT3CART showed anti-leukemia activity, potentially reducing antigen escape [65].

FLT3-targeting CAR NK cells have also shown significant promise in AML treatment. Frozen allogeneic NK cells engineered with FLT3 CAR and IL-15 (FLT3 CAR_sIL-15 NK) demonstrated enhanced cytotoxicity and increased IFN-γ secretion against FLT3 + AML cell lines. These CAR NK cells improved survival in xenograft AML models without impacting healthy blood or hematopoietic cells. These findings suggest that FLT3-targeted CAR NK cells could provide an off-the-shelf AML therapy with sustained persistence, offering a potentially effective and convenient treatment option [66].

Another study explored FLT3-CAR macrophages, showing that FLT3 can polarize M2-like leukemia-associated macrophages (M2-LAM) and impair phagocytic function. FLT3-CAR macrophages restored phagocytic activity, reducing leukemic burden and improving survival in AML xenografts. Pexidartinib treatment further sensitized FLT3-ITD + MOLM-13 cells to FLT3L-CAR macrophages, suggesting a potential preclinical strategy for FLT3-ITD + AML [67].

IL-1RAP is another promising marker for AML, as it is highly expressed on leukemia stem cells (LSCs) and AML blasts but absent on HSCs, making it an ideal target. Trad et al. developed IL-1RAP CAR T-cells, demonstrating significant anti-tumor activity in vitro and in vivo. These CAR T-cells showed efficient infiltration, proliferation, and persistence in the blood, bone marrow, and spleen. The therapy exhibited potential for treating both initial and relapsed AML, reducing tumor burden and extending survival, highlighting its therapeutic promise [68].

Based on these findings, targeting CSCs with CAR-based therapies, such as CAR T-cells, CAR NK cells, and CAR M, demonstrates this treatment approach’s feasibility and potential effectiveness in cancer. Despite ongoing challenges, extensive clinical studies evaluating the performance of CAR T-cells against CSCs have generally yielded positive results, further supporting the promise of this therapeutic strategy (Table 1).

#### Chronic myeloid leukemia (CML)

Like the AML, various CSC surface markers expressed on CML have been targeted by CAR-based structures, such as CD44, CD47 (IAP), CD52 (Campath-1), CD56 (NCAM), CD90 (Thy-1), CD93, CD114, CD135 (FLT3), CD295 (LEPR), CD25, TIM3, CD26, and IL1RAP [69]. The studies showed the efficacy and safety of anti-CSC CAR-based treatment in CML, for instance, the CD25 as an LSC marker related to the progression of tumors in CML, targeted by anti-CD25 CAR NK cells. These specific CAR NK cells indicated anti-tumor function on CD25 CML cells in vivo and prolonged survival time in many mice models [70]. Recently, anti-CD-26 CAR macrophages were designed to target CML LSCs. A study indicated that anti-CD-26 CAR macrophages improve survival and decrease invasion of CML in vivo and in vitro, unveiling the applicability of CAR macrophages in treating hematological malignancies [71]. Despite all the above results, only some of the fundamental antigens in CML have been targeted, and information in this area still needs to be available. Due to the close similarities between CML and AML, CML may also yield positive results in tumor removal, and similar outcomes could be achieved by targeting its stem cells.

#### Multiple myeloma (MM)

Several CSC markers, such as ALDH, CD138, CD24, BTK, RARα2, ROS, CD44, and CD19, have been recognized in the MM manner [72]. Among the various stem cell antigens, CD138 and CD19 have been targeted by CAR T-cells. However, CAR NK cells, CAR M, and CAR NKT cells have yet to be employed to target multiple myeloma stem cells. In a clinical trial, CAR T-cells targeting CD138-positive MM were tested in five patients. The results showed that four out of five patients achieved stable disease for over three months, while one patient with progressive plasma cell leukemia experienced a reduction in myeloma cells in her peripheral blood. Furthermore, no intolerable toxicities or safety concerns were reported, indicating that this approach could be a viable therapeutic strategy for targeting multiple myeloma stem cells [73]. Despite the functional outcome of CAR T-cell therapy, other CAR-based therapies, particularly CAR NK cells, must be evaluated.

#### The other hematological tumors

Studies on targeting CSCs with CAR-based therapies in acute lymphoblastic leukemia (ALL) and chronic lymphoblastic leukemia (CLL) and also identifying markers that exhibit CSC characteristics in CLL are limited. Consequently, leukemia-initiating cells (LICs), which share characteristics with CSCs, are still detectable. This underscores the need for further research to analyze these cells. Certain studies have identified markers indicative of CSC characteristics in B-cell ALL. For instance, markers such as immature CD34^+^CD19^−^ or mature CD34^+^CD19^+^, CD133^+^CD19^−^ and CD133^+^CD38^−^ cells in childhood B-cell ALL are associated with LICs. Moreover, markers such as CD34 + CD7 + and CD7 + CD1a in T-cell ALL have been shown to exhibit LIC characteristics. Therefore, the efficacy of CAR-based therapies in targeting LICs should be evaluated in future research [74]. Also, some studies recruit CAR T/NK cells to target lymphomas, and some of them achieved promising outcomes in the first phases. Nonetheless, as we know, no study targets the lymphoma stem cell markers with CAR-based cells [75].

The limited studies on LSC markers in CAR-based therapies may be attributed to several factors. First, the existence and characterization of lymphoma stem cells remain poorly defined, unlike solid tumors [76]. Many lymphomas originate from mature B or T cells, making it challenging to distinguish a distinct stem-like subpopulation with self-renewal capabilities [76]. Additionally, lymphomas are highly heterogeneous, with diverse subtypes such as DLBCL, FL, and HL exhibiting various biology and antigen expression profiles. This complexity makes it difficult to identify universal LSC markers that CAR-based approaches can effectively target [76, 77].

### Solid tumors

#### Glioblastoma

Several markers for glioblastoma stem cells (GSC) have been discovered and isolated, including CD133, B7-H3, GRP78, GD2, EGFRvIII, SOX-2, and HMG box, L1CAM (CD171), CD15, CD44, CD34, CD36, CD49, Musashi-1, and Nestin [78, 79]. To eliminate these markers, several studies have utilized CAR-based therapies, such as CAR T-cells, CAR NK cells, and CAR M, to target them both in vitro and in vivo. This tumor is much attention to treatment with CAR-based therapy due to the high invasion capability and more relapse/recurrence after standard of care (SoC).

Regarding the CAR T-cells, many investigations have been done in this setting in vivo and in vitro, targeting various GSCs and demonstrating excellent outcomes in the anti-GSC CAR-based treatment. For instance, the studies in the anti-GD2 CAR T-cell setting showed repeating the high efficacy in this manner, like have been showing that utilizing this CAR in co-culture with glioblastoma cell lines and patient-derived primary glioblastoma cells to evaluate the cytokines released, can increase expression of these cytokines in multiple cell lines. Despite the expression of TGF-β in the cultures, it did not influence anti-tumor activity. Overall, the current study confirmed the safety and reliability of anti-GD2 CAR T-cells in demonstrating anti-tumor activity both in vitro and in vivo [80]. Indeed, in clinical trials, the fourth generation of anti-GD2 CAR T-cells has been evaluated in eight patients. The current study showed that all patients survived for over six months, although three experienced progressive disease. It was observed that after the infusion of TRUCK alone or combined with intracavitary administration, all patients had elevated levels of TNF-α and IL-6, illustrating its contribution to antigen loss and the recruitment of an immune response in the TME. Moreover, the study demonstrated the safety and persistence of TRUCK with no adverse effects [81].

These remarkable results have been replicated by targeting other CSC markers through CAR T-cell therapy. For example, one study demonstrated that second-generation anti-B7-H3 CAR T-cells effectively inhibited tumor growth in vitro and in vivo. In this study, anti-B7-H3.CD28 and anti-B7-H3.4-1BB CAR T-cells successfully eradicated glioblastoma multiforme stem cells in culture. However, anti-B7-H3.CD28 CAR T-cells secreted higher levels of cytokines, including IL-2 and IFN-γ, compared to the anti-B7-H3.4-1BB CAR T-cells [82]. As with other GSCs, several studies have focused on Epidermal Growth Factor Receptor variant III (EGFRvIII), associated with stemness and GSCs. Targeting EGFRvIII is one of the most commonly considered CAR-based therapies in GBM, showing different outcomes depending on the patients, cell lines, and animal models [83, 84]. Another example involves anti-GRP78 CAR T-cells, which were investigated for their efficacy in targeting GRP78 antigens. This study demonstrated that the CAR T-cells effectively targeted GRP78, as evidenced by the secretion of IFN-γ in the tumor microenvironment both in vitro and in vivo. These findings suggest that targeting GRP78 represents a promising strategy for cancer therapy, potentially improving tumor control and immune response [85]. Finally, targeting NKG2D, which is upregulated in GSCs, has proven to be an effective strategy. Second-generation anti-NKG2D CAR T-cells have demonstrated the ability to release high levels of cytokines, such as perforin and granzyme B, to eliminate GBM cells and GSCs. These CAR T-cells exhibited impressive persistent anti-tumor activity in vitro and in vivo without showing any toxicity associated with the treatment. This suggests that targeting NKG2D could be a promising approach for treating GBM and its stem cells [86].

Despite the success of CAR T-cells in treating tumors, a significant barrier remains: CAR T-cell therapy often cannot sufficiently penetrate solid tumors. To address this challenge, CAR M has been designed [18]. In one study, researchers injected a hydrogel containing nano porters into the cavity left after glioma tumor resection. These nano porters within the hydrogel carried genes encoding CARs targeting the CD133 antigen in GSCs. The nano porters then delivered these genes to the nuclei of macrophages and microglia surrounding the cavity, producing CAR M/microglia (CAR M). These CAR M could eliminate tumors by recruiting NK cells, T-cells, and DCs to the GSCs. They also engulfed and cleared CD133-positive glioma cells, acting as antigen-presenting cells to induce an adaptive immune response, which prevented tumor regrowth for the long term in vivo. Furthermore, the combination of CAR M with a CD47 antibody had a synergistic effect on the tumors, resulting in considerable anti-tumor activity [87].

Indeed, CAR NK cells, like CAR T-cells, have proven effective in targeting GSCs. B7-H3 CAR NK cells were engineered to target GBM cell lines in a study. However, the efficacy of these CAR NK cells was hindered by TGF-β released by the glioblastoma cells. To overcome this challenge, the researchers co-transduced B7-H3 CAR NK cells with a dominant-negative TGF-β receptor (DNR) to protect the CAR function from TGF-β-mediated suppression. The results demonstrated that the co-transduced anti-B7-H3 CAR NK cells, with the protection provided by the DNR, effectively eliminated glioblastoma tumors by secreting IFN-γ in the tumor microenvironment. This approach highlights the potential of combining CAR NK cell therapy with TGF-β inhibition to improve anti-tumor efficacy [88]. As a result, B7-H3 could be considered a therapeutic target for GSCs. Other studies have presented CAR NK cells as an off-the-shelf application that is not limited to individual patients. An investigation proposed the combinational immunotherapy of anti-GD2 CAR NK92 and PBMC-derived induced neural stem cells (iNSCs) expressing HSV-TK, targeting GD2 antigens in GBM both in vitro and in vivo. PBMC-derived iNSCs expressing HSV-TK exhibited the tumor-tropism by migrating toward GBM cells. After reaching the tumors, they exerted anti-tumor activity, particularly in the presence of ganciclovir, which activates HSV-TK genes to eliminate tumor cells through the bystander effect. Moreover, the current study combined the anti-GD2 CAR NK92 and significantly amplified the activation of anti-tumor effects [89].

A recent study utilized anti-NKG2D-CAR NK-92-cells by adding a potent form of IL-15 (RD-IL15) and bispecific engagers. These modifications enabled the NK cells to incorporate RD-IL15 (NKAR_RD-IL15-NK-92) and simultaneously engage with both NKG2D ligands and TAAs, such as Epidermal growth factor receptor (EGFR) or ErbB2 (HER2), in GSCs. This combination therapy enhances the NK cells’ ability to recognize and destroy cancer cells expressing these antigens and could eliminate both NKG2D + and NKG2D- tumor cells. Furthermore, anti-NKG2D-CAR NK-92-cells demonstrated a cytotoxic effect and the ability to prevent immune escape in an in vitro environment. Additionally, the IL-15 secreted by the NKAR-RD-IL15 NK-92-cells increased their cytotoxicity and enhanced the proliferation of cytotoxic T lymphocytes (CTLs) present in the environment [90].

Consequently, according to numerous studies discussing the targeting of GSCs by various types of CAR-based therapies, it is essential to evaluate the targeting of CSC markers across all age groups. CD133 and CD49f are expressed in both adult and pediatric groups; however, targeting these antigens may lead to different outcomes due to the distinct biological pathways and mechanisms in pediatric tumors compared to adult tumors [79].

#### Prostate

Targeting CSCs with CAR-engineered cells has shown potential, using antigens like prostate stem cell antigen (PSCA), EpCAM, and B7-H3. Notably, CD117 is associated with heightened prostate cancer (PCa) aggressiveness by fostering CSC traits and resistance to tyrosine kinase inhibitors [91].

CAR-modified T-cells and NK cells have shown promising results in targeting CSCs, while macrophages and NKT-cells remain underexplored. For example, second-generation anti-EpCAM CAR T-cells, engineered with the co-stimulatory CD28, significantly improved survival in EpCAM-expressing PCa models both in vitro and in vivo. This suggests that CAR T-cell therapies targeting specific antigens like EpCAM hold the potential for enhancing treatment outcomes, though further research into other immune cells, such as macrophages and NKT-cells, could expand therapeutic possibilities [92]. Additionally, anti-B7-H3 CAR T-cells, combined with fractionated irradiation (FIR) to boost B7-H3 expression, have proven effective in eliminating CSCs, potentially addressing antigen loss during therapy [93]. Thus, Dual-targeting CAR approaches, like EpCAM and B7-H3 bispecific CAR T-cells, could offer enhanced selectivity against CSCs.

Another study revealed that utilizing γδ-Enriched CAR T-cell therapy against PSCAs could attenuate established tumors in bone metastatic castrate-resistant prostate cancer (CRPC) in vitro and in vivo, which this function was amplified by prescribing zoledronate. γδ T-cells promote CAR T-cell cytotoxicity and facilitate the identification of phosphoantigens accumulating in the bone [94]. Another study demonstrated that minicircle DNA-Engineered CAR T-cells, generated by minicircle DNA (mcDNA), represent a promising approach targeting PSCAs for eliminating growth tumors in mice. Besides their cytotoxicity capacity, anti-mcDNA-PSCA CAR T-cells exhibit high persistence, allowing them to overcome exhaustion and senescence in vitro and in vivo [95].

CAR NK cells targeting CSC markers have also shown promising outcomes. For instance, a study demonstrated that universal off-the-shelf dual CAR NK92-cells, engineered to target prostate-specific membrane antigen (PSMA) simultaneously and PSCA, effectively released cytokines such as IFN-γ, perforin, and granzyme B in response to prostate cancer in vivo. Additionally, this dual-targeting approach helped reduce the risk of on-target, off-tumor effects, enhancing both the safety and efficacy of the treatment [96]. Another research indicated that utilizing NK cells modified with DAP12-based CARs to activate NK cell lines can be a promising approach against PSCA-expressing tumors. For instance, the anti-PSCA-DAP12 CAR improves the cytotoxic abilities of the YTS NK cell line against tumor cells expressing the PSCA antigen in vitro and in vivo [97].

Based on the investigation into CSC-targeting with CAR NK and T-cells, this treatment method had promising results as a target antigen. However, further clinical trials are required to evaluate the efficacy of CAR-based therapies in targeting prostate CSCs, and maybe the use of other immune cells can be beneficial in this manner.

#### Kidney and osteosarcoma

Several markers have been identified for both renal and osteosarcoma CSCs, including CD105, CD133, and CD44 in the kidney, and CD133, CD117, Stro-1, CD271, ALDH, and Oct4 in osteosarcoma [98, 99]. Furthermore, research has shown that DNAJB8, a member of the HSP40 family of proteins, is significantly expressed in renal CSCs and plays a protective role in their survival. This expression has also been observed in osteosarcoma cell lines. Despite the abundance of CSC markers, CAR-based therapies targeting them remain limited, and only CAR T has been examined in this setting. A study was aimed to address this gap by developing a bispecific second-generation CAR T-cell named B10 CAR T-cell. This CAR targets both HLA-A24:02 (a specific human leukocyte antigen) and a peptide derived from DNAJB8 (DNAJB-143) [100]. The in vitro and in vivo study demonstrated the effectiveness and functionality of B10 CAR T-cells, suggesting their potential as a promising candidate for immunotherapy. This study highlights DNAJB8 as a promising target, but further research is necessary to identify and validate additional CSC markers [100]. Additionally, optimizing B10 CAR T-cell function and evaluating its efficacy in preclinical models will be crucial before clinical trials can be initiated.

#### Breast cancer

Several markers for breast cancer stem cells (BCSC) have been recognized, such as LGR5, CD105, CD47, CD166, c-Met, ROR1, CD133, and others [101]. Some markers have served as targets for various CAR-based therapies. While studies on CAR NK and CAR T-cells have explored various CSCs, research on CAR M and CAR NKT-cells targeting BCSCs still needs to be explored.

A study demonstrated that anti-GD2 CAR T-cells can eliminate BSCSs in vitro and in vivo. The mentioned CAR is produced by a novel ScFv obtained from the anti-GD2 monoclonal antibody Dinutuximab and incorporates the co-stimulatory molecule 4-1BB, which works together to prevent exhaustion. Anti-GD2-CAR T-cells can prevent metastasis, even if the tumors separate from the primary tumor [28, 102]. Another study showed that second- and third-generation anti-TEM8 CAR T-cells, which target TEM8 markers identified as BCSC markers in triple-negative breast cancer, can neutralize the tumor-associated vasculature and BCSCs in vitro and in vivo. Anti-TEM8 CAR T-cells secrete IFN-γ and IL-2 around the TEM8^+^ TNBC and kill this tumor [103]. Another study demonstrated that the anti-tissue factor CAR NK cells can help abolish TNBC cells by targeting tissue factor (TF), which can be expressed in BCSCs. Moreover, the results can be enhanced by combining TF-targeting antibody-like immunoconjugate (ICON). In vitro and in vivo studies, the ICON was updated to have advanced ADCC against TNBC cells compared to the original ICON [104]. While CAR-based therapies targeting BCSC markers showed promise, further research is needed to explore these and other CAR designs for BCSC eradication.

#### Pancreas cancer

In pancreatic cancer, markers such as CD133, CXCR4, DCLK1, c-MET, ABCG2, and Lgr5 play a role [105]. However, few CAR-based therapies have been developed to target CSCs despite these markers. Currently, CAR T-cell therapy is the only approach that has been utilized.

The CEACAM7 marker is primarily expressed on the apical surface of pancreatic ductal cells and epithelial cells in the adult colon, with minimal to no expression in other gastrointestinal tissues. This selective expression makes CEACAM7 an ideal target for CAR T-cells, as it could help minimize toxicities. Raj et al. found that CEACAM7 is highly expressed in CSCs from pancreatic ductal adenocarcinoma compared to normal cells. Consequently, they developed second-generation anti-CEACAM7 CAR T-cells with the co-stimulatory 4-1BB domain to target this marker. These CAR T-cells demonstrated anti-tumor solid activity without inducing toxic side effects, highlighting their potential as a safer therapeutic option [106]. The pancreas also expresses another antigen, PSCA, which is expressed in the prostate and the normal pancreas tissue and overexpressed in pancreatic cancer cells [107]. This antigen is targeted by freeze-thawed, off-the-shelf CAR NK cells expressing soluble IL-15 after both in vitro and in vivo studies. The CAR NK cells could persist for over 90 days in mice with metastatic pancreas and showed a reliable response with no observed toxicity in both in vitro and in vivo experiments [108]. These findings suggest that anti-PSCA CAR NK cells hold promise as a therapeutic option for pancreatic cancer and merit further investigation. Another study explored the use of human iPSC-derived CAR M engineered to express IL-15 for enhanced immune activity and a truncated EGFR as a safety “suicide switch.” These CAR M cells were designed to target PSCA on pancreatic cancer cells to create a cryopreserved, allogeneic “off-the-shelf” therapy. Results were promising, demonstrating effective anti-tumor responses in both in vitro and in vivo models, with no signs of CRS or toxicity in vivo, underscoring the potential of CAR M therapy as a safe and effective treatment option [109].

#### Colorectal cancer

Several specific markers, such as CD133, CD44, CD166, EpCAM, Lgr5, and BMI1, are known to identify colorectal CSCs [110]. To target these CSCs, CAR T-cell and CAR NK cell therapy have been employed, whereas other CAR-based therapies have not yet been utilized.

CD133 marker was targeted by two models of CAR-based therapies, including CAR T-cells and CAR NK cells, in clinical trials and laboratory tests, respectively. The outcome of CAR T-cells in targeting CD133-positive colorectal cancer showed a safe and feasible immunotherapeutic approach (Table 1). In another study, researchers developed anti-CD133 CAR NK92 cells (CAR133-i502-NK92) engineered to secrete the TLR5 agonist CBLB502 at the tumor site. This modification enhanced proliferation, cytokine production, and antitumor activity in vitro compared to standard anti-CD133 CAR NK92 cells. These engineered cells effectively targeted CD133 + and mixed CD133+/CD133- colon cancer cells in mouse models and were well tolerated in vivo. The secreted CBLB502 activated endogenous immune cells, aiding tumor eradication by enabling CAR133-i502-NK92 cells to eliminate CD133 + cells while indirectly targeting CD133- cells directly. This dual-targeting approach presents a promising therapeutic strategy for colorectal cancer [111].

Two studies examined the therapeutic potential of targeting the EpCAM molecule, commonly expressed in various digestive system cancers, using different CAR-modified immune cell strategies. The first study utilized the anti-EpCAM CAR T-cell against CRC and gastric cancer and reported the feasibility and safety of CAR T-cell therapy (Table 1) [112]. The second study used the second-generation anti-EpCAM CAR-NK-92-cell therapy to target CRC. These cells exhibited strong cytokine release and cytotoxic activity in vitro, and combination therapy with regorafenib led to enhanced tumor growth suppression in a mouse model [113]. The studies suggest that EpCAM-targeted CAR therapies, whether using T or NK cells, show promise in treating EpCAM-positive cancers, with combination treatments offering the potential for greater therapeutic effectiveness.

A study assessed Programmed cell death-ligand 1 (PD-L1) expression in colorectal CSCs and non-CCSCs and designed a dual immunotherapy strategy involving anti-PD-L1 CAR-T-cells and a CCSC-DC vaccine. Anti-PD-L1 CAR-T-cells were engineered to target PD-L1 molecules on CCSCs, while the CCSC-DC vaccine was made from CCSC lysates to stimulate T-cells. ALDH1-positive CCSCs were abundant in patients’ tumor samples and cell lines, and these CSCs showed higher PD-L1 expression than non-CCSCs. While individual treatments with anti-PD-L1 CAR-T-cells or the CCSC-DC vaccine only had moderate effects in reducing tumors, the combination therapy significantly enhanced cancer cell destruction and reduced tumor burden in mouse models [114].

Another study demonstrated that anti-CD6 CAR T-cells targeting CD6 exhibited cytotoxic effects, secreted IFN-γ around the tumors, and eliminated them in vitro [115]. As previously mentioned, CD166 is one of the markers of colorectal CSCs and binds to CD6 through the SCRC3 domain. Therefore, anti-CD6 CAR T-cells targeted CD166 markers and lysed them by secreting IFN-γ cytokines. Interestingly, while this CAR was designed to target CD6 ligands, CD166 and CD318, it predominantly targeted CD166 instead of CD318.

LGR5, a marker found on CSCs and involved in Wnt/β-catenin signaling, plays a critical role in CRC tumor initiation and metastasis and is found in CSC. A study designed a CAR to target the extracellular domain of LGR5 and validated its on-target specificity and effectiveness. In laboratory tests, CAR-T-cells exhibited potent cytotoxicity and specific IFNγ production against CRC and neuroblastoma cell lines. They were further tested for safety by comparing responses to normal human tissues from organs such as the colon, liver, and heart, showing minimal off-target effects. Moreover, these CAR-T-cells also maintained a central memory phenotype, which is critical for long-term anti-tumor activity [116]. These studies highlight LGR5 as a promising therapeutic target, with anti-LGR5 CAR-T-cells showing potential for future clinical trials in CRC treatment. Moreover, by screening a cohort of 472 patients with primary tumor biopsies, it was found that approximately 60% of the tumors had moderate to high levels of LGR5 expression, with higher levels correlating with more advanced and higher-grade tumors. Additionally, LGR5 expression in metastatic lesions, particularly in liver and lymph node metastases, is associated with primary tumor expression [117]. Therefore, LGR5-positive patients may benefit from the LGR5-positive CAR T-cells.

#### Gastric cancer

Several gastric CSC markers have been isolated, including CD44, ALDH1, CD90, CD71, and EpCAM [118]. Currently, CAR T-cell therapy is the only approach used against gastric CSC, and further investigation into other CAR-based therapies, such as CAR NK, CARM, and CAR NKT, is needed.

Unlike normal tissues, certain tumors’ B7-H3 (CD276) marker is upregulated. A study found that B7-H3 expression is elevated in gastric cancer and is linked with stemness markers, including Sox2, Prom1, NGFR, and THY1, suggesting its potential as a CSC marker. This study employed anti-B7-H3 CAR T-cells capable of secreting IL-2 and IFN-γ in the tumor microenvironment, effectively eliminating stem-like tumor cells in vitro and in vivo. No pathological damage was observed in critical organs such as the treated mice’s heart, liver, lungs, stomach, spleen, and kidneys, underscoring the therapy’s safety [119]. Another study demonstrated anti-human epidermal growth factor receptor 2 (HER2) CAR T-cells. HER2 (CD340), a protein encoded by ERBB2, maintains the stem cell subpopulation of gastric cancer and is upregulated in gastric CSCs [120]. The anti-HER2 CAR T-cells targeted HER2 + gastric cancers in vitro and in vivo. They released cytokines, including IFN-γ, IL-2, IL-4, GM-CSF, and TNF-α, around the tumors, aiding the anti-tumor activities of CARs. However, despite their effectiveness, high levels of cytokines can lead to CRS.

Another study indicated that anti-CD133 CAR T-cells and Cisplatin exhibited cytotoxicity against CD133 + gastric CSCs in vitro and in vivo. The CAR released cytokines such as IL-2, IFN-γ, GM-CSF, and TNF-α, which are implicated in CRS. In this study, Cisplatin increased the expression of CD133 in CSCs, aiding CAR T-cells in targeting these antigens and overcoming the antigen loss barriers [121]. Increased CD133 expression induced by Cisplatin can help address antigen loss barriers.

#### Lung cancer

Lung cancer, recognized as one of the most lethal malignancies, is categorized into small-cell lung cancer (SCLC) and non-small-cell lung cancer (NSCLC). Certain CSC markers are uniquely associated with SCLC, while others are specific to NSCLC. Additionally, some markers are common to both subtypes. The CSC markers specific to NSCLC include CD44, CD90, CD117, CD133, CD166, EpCAM, ABCG2, FZD, CXCR4, ALDH, SOX2, OCT4, NANOG, and BMI1. Among these CSC markers, CD44, CD90, CD133, CD166, EpCAM, ABCG2, ALDH, SOX2, OCT4, and BMI1 are common to both NSCLC and SCLC. Some CSC markers, such as CD87/µPAR, PODXL-1, and PTCH, are specifically associated with SCLC [122]. Among CAR-armored cells, only CAR T-cells have been employed to target the CSC markers in both subtypes of lung cancer. The anti-CSC CAR-based therapy can show great drama in various lung cancers, but some challenges have remained. The different anti-CSCs CAR T-cells like anti-cell surface glucose-regulated protein 78 (csGRP78) CAR T, anti-PSCA and MUC1 CAR T, anti-CD47 CAR T, anti-PD-L1 CAR T, in NSCLC, have proven anti-tumor studies against tumor cells in vivo and in vitro [123, 124]. For instance, a recent study utilized anti-csGRP78 CAR T-cells to assess their function in NSCLC. The csGRP78 is expressed on various hematological and solid tumors but is limited to the interior of normal cells and shows CSC features. They constructed GRP78-binding peptide Pep42 CAR T-cells to recognize csGRP78^+^ cells in NSCLC and CSCs. These CAR T-cells eliminated csGRP78^+^ cell lines, exhibited potential anti-tumor activity, and elevated levels of IFN-γ in vitro. Additionally, they evaluated the function of these CAR T-cells in vivo and observed increased tumor apoptosis and enhanced tumor infiltration while sparing normal tissues [125]. Furthermore, CD47 is a marker that plays an important role in CSCs, with its mRNA expression level correlated with CD133 mRNA expression level in adenocarcinoma, squamous cell carcinoma, and SCLC [126]. A study employed the anti-CD47 CAR T-cells targeting CD47^+^ in NSCLC cell lines. The researchers observed that these cells inhibited tumor growth, elevated IFN-γ and IL-2 levels, and reduced the expression of genes involved in metastasis, such as β-actin, calreticulin, and cyclooxygenase 2 [127]. As a result, further investigations, particularly clinical trials, are needed to evaluate the efficacy of CAR T-cells on various CSC markers in NSCLC. Moreover, in vitro and in vivo studies on the function of CAR NK cells may help identify promising approaches for treating NSCLC.

Research has explored CSC markers in SCLC, highlighting a study on third-generation anti-AC133 CAR T-cells targeting CD133 in human and mouse models. While these CAR T-cells significantly reduced tumor burden and improved survival, they didn’t eliminate tumors. However, combined with a CD73 inhibitor and an anti-PD-1 antibody, the therapy achieved cures in 25% of models, effectively targeting chemo-resistant tumor stem cells and potentially inducing long-term remission without causing bone marrow failure or GvHD [128].

The following studies reviewed markers common to NSCLC and SCLC, including PTK7, EpCAM, and CD44. A study designed the anti-PTK7 CAR T-cells to eliminate this tumor both in vitro and in vivo. The anti-PTK7 CAR T-cells demonstrated a high potential against PTK7^+^ tumors and elevated levels of IFN-y and IL-2 without affecting normal tissue due to less expression than cancer tissues. Moreover, anti-PTK7 CAR T-cells exhibited the suppression of PD-1 due to the release of granzyme B [129].

No direct studies have targeted EpCAM in primary lung cancer, but some research has examined EpCAM-positive brain metastases from lung cancer. One study evaluated anti-EpCAM CAR T-cell therapy for these metastases and its combination with anti-PD-1 antibodies. In a mouse model, CAR T-cells were injected into the brain, showing effective tumor infiltration, growth inhibition, and extended survival. However, adding anti-PD-1 antibodies did not enhance CAR T-cell persistence or tumor reductio [130]. The study concludes that while CAR T-cell therapy holds promise for brain metastases, combining it with anti-PD-1 treatment does not provide additional benefits. New strategies are needed to improve its effectiveness against solid tumors.

One study employed anti-CD44v6 CAR T-cells against lung adenocarcinoma in mice models (MR232). Mice were challenged with tumor cells and treated with anti-CD44v6 CAR T-cells at a lower dose to mimic potential clinical applications. However, in the more aggressive lung adenocarcinoma model, anti-CD44v6 CAR T-cells did not initially control tumor growth, while increasing the CAR T-cell dose led to better tumor control, highlighting that the tumor’s aggressiveness and growth rate can influence the required CAR T-cell dosage for effective treatment [131].

#### Hepatocellular carcinoma

Several hepatocellular carcinoma (HCC) CSCs have been identified and exhibit the potential to be targeted by CAR-based therapies, including CD133, CD44, EpCAM, CD90, Ov6, and CD13 [132]. Among CAR-based therapies, only CAR T-cells have been chosen to target HCC CSCs. CD133 and CD44 are the markers reported in targeting by CAR T-cells. The CAR T-cell can achieve excellent results in the anti-CSC in the HCC setting. For example, Yang et al. generated anti-CD133 CAR T-cells that secrete a PD-1 ScFv blocker using a unique method called the Sleeping Beauty transposon system, which utilizes minicircle vectors. This approach is more cost-effective than using viral vectors. The combination therapy demonstrated higher anti-tumor activity than CAR T-cells alone and mock T-cells in male patients with advanced HCC. This outcome was confirmed both in vitro and in vivo [133]. Also, anti-COG133 CAR T-cells have been generated with nonviral mcDNA to target CD133 and GPC3 antigens. This structure had significant tumor suppression without affecting normal tissues in mice [134]. Furthermore, a clinical study reported the targeting of CD133 by CAR T-cells, with the results shown in (Table 1). Considerably, due to the liver toxicity of preconditioning drugs like Cyclophosphamide and Fludarabine, lymphodepletion therapy is not prescribed in these clinical trial studies [135, 136]. Another study developed an innovative platform using non-viral mcDNA to produce anti-CD44 CAR T-cells and evaluated their efficacy in laboratory and animal studies. After seven days, the mcDNA-CD44-CAR gene was successfully introduced into human T-cells with over 50% transfection efficiency. These CAR T-cells exhibited similar properties to regular T-cells but demonstrated enhanced antitumor activity, both in vitro and in vivo, against CD44-positive HCC tumors. In xenograft mouse models, anti-CD44 CAR T-cells effectively inhibited tumor growth, with no significant side effects observed, suggesting that this approach holds promise for treating CSC-related tumors [137]. Based on studies investigating the functions of various CAR T-cells targeting different cancers and their outcomes with minimal toxicity, CAR T-cells appear to be a promising therapeutic approach against HCC CSCs. Furthermore, a clinical study indicated that patients with a lower tumor burden experienced more extended periods of stable disease. These findings support the idea that if the therapy is administered earlier, after surgery, patients may experience higher survival rates [135]. Consequently, further studies are necessary to investigate this possibility. Additionally, more research is required to evaluate the efficacy of other CAR armored immune cells in targeting hepatocellular cancer stem cells.

#### Cholangiocarcinoma

Various markers in cholangiocarcinoma have been identified as CSCs, including CD133, CD44, CD24, EpCAM, ALDH, SOX2, NANOG, and OCT4 [138]. Among markers, as we know, only CD133 has been evaluated and targeted by CAR T-cell therapies. A study utilized fourth-generation anti-CD133 CAR T-cells targeting CD133^+^ tumor cell lines in cholangiocarcinoma, which exhibited an anti-tumor response, as well as an increase in IFN-γ and TNF-α around the tumors [139]. Another study investigated second-generation anti-CD133 CAR T-cells combined with an anti-PD-L1 ScFv to target CD133 + tumors. This tandem CAR showed substantially higher long-term cytotoxicity than anti-CD133 CAR T-cells, decreasing PD-1 expression in vitro. However, in the short term, the tandem CAR did not exhibit a significant difference in effectiveness compared to CAR monotherapy [140]. Based on the study’s outcomes and the obstacles encountered, further research is needed to evaluate the function of anti-CD133 CAR NK cells in targeting cholangiocarcinoma CSCs.

#### Ovarian cancer

Several markers are recognized as ovarian CSCs markers, including CD133, CD44, ALDH1, CD24, CD117, CD105, CD106, EpCAM, Thy-1 (CD90), SOX2, Nestin, and SSEA1 [141]. These identified markers are suitable targets for CAR-based therapies to eradicate tumors. Among CAR-based therapies, CAR T-cells and CAR NK cells have been employed against ovarian CSCs, while other CAR-armored cells, such as CAR M and CAR NKT-cells, have not been utilized. A study engineered a third-generation CAR T-cell targeting the EpCAM antigen, incorporating co-stimulatory and activation domains. The CAR was introduced into T-cells using lentiviral transduction. The results showed that the anti-EpCAM CAR T-cells effectively killed cancer cells and triggered cytokine release in vitro. In animal models, these CAR-T-cells also led to significant tumor shrinkage, suggesting that anti-EpCAM CAR-T therapy could be a promising new approach for ovarian cancer treatment [142]. A study utilized the codon-optimized third-generation anti-CD24 CAR NK against CSCs, which exhibited a high antitumor effect on CD24^+^ ovarian cancer cell lines and CD24^−^ cell lines that were modified to express CD24 using a CD24 transmembrane protein lentivirus. Moreover, to reduce the risk of off-target effects in vivo, the authors employed the dual CD24 and MSLN CAR to target both tumor markers simultaneously. The outcome was highly cytotoxic, similar to anti-CD24 CAR NK cells [143]. Also, a study incorporated NK92-cells into a third-generation CAR for targeting CD133^+^ tumors. This study indicated the fatal effects of these CAR NK92-cells on CD133 + cells in vitro. Additionally, to achieve better anti-tumor response and overcome recurrence, they combined specific CAR NK92-cells with Cisplatin, demonstrating a stronger killing effect than monotherapy alone. The authors showed that using sequential therapy, administering NK92-cells after prescribing Cisplatin, promoted the efficacy of anti-CD133 CAR NK cells. However, this effect was not observed in CD133-negative tumor cells [144]. Targeting CD44v6, a variant of CD44 protein, is another marker that CAR-based therapies can target. In one study, anti-CD44v6 CAR T-cells were utilized to target solid tumors, particularly ovarian adenocarcinoma and lung cancer. The antitumor effects of these CAR T-cells were assessed in an ovarian carcinoma model (IGROV-1-luc) in mice. Mice were challenged with tumor cells and treated with a lower dose of anti-CD44v6 CAR T-cells to simulate clinical application. Results showed that a single infusion of anti-CD44v6 CAR T-cells significantly reduced tumor growth and prolonged survival compared to control groups, highlighting the potential of this therapy for ovarian carcinoma [131]. Another study employed anti-CD44 CAR NK cells to target CD44^+^ cell lines. The results indicated that the efficacy of anti-CD44 CAR NK cells was significantly enhanced when combined with Cisplatin administered simultaneously, as opposed to sequentially, which contrasts with the previous study’s findings. However, consistent with the previous study, anti-CD44 CAR NK cells did not demonstrate effectiveness against CD44-negative tumor cells [145]. Indeed, while CAR-based therapies have shown promising results in targeting CSCs, particularly in preclinical models, the limited number of studies calls for more extensive research. Further preclinical and clinical studies are necessary to understand better these therapies’ long-term efficacy, safety, and potential challenges.

Table 2 mentions the clinical trial, case report, and case series targeted by CAR-based treatment after prescribing the precondition drugs. Each explains the result of this immunotherapeutic approach and how it has been managed.

**Table 2:** Clinical trial, case report, and case series based-anti-CSC CAR treatment

CAR structure	Cancer Type	Precondition drugs	Adverse effects	Efficacy	Ref.
Donor-Derived CD123-CAR T-cell	AML	Therarubicin, Teniposide, Flu, and Buslfan	developed grade 4 GvHD and CRS.	CR with incomplete blood count recovery and myeloid implantation.	[146]
UniCAR-T-CD123	r/r AML	Flu/CY	The structure was safe and tolerable. Most patients reached hematological recovery. Indeed, most patients experienced mild to moderate toxicities, including grade 1–2 CRS, and one case of grade 3 CRS, and one of them had ICANS.	Two patients had CR, and four had PR.	[147]
CD123-CAR T-cell	r/r AML	Flu/Cy or Flu/Cy and Alemtuzumab	Of 16 patients, 15 developed CRS (3 with G4+), and 1 had G3 ICANS.	Four patients showed CAR-T activity: 1 MLFS, 1 SD in the Flu/Cy arm, and 1 SD, 1 MRD-negative CR (8 months) in the Flu/Cy + A arm, which had better lymphodepletion and expansion	[148]
RNA CD123-CAR T-cell	r/r AML	Cy	RNA CART123 therapy was safe, with no treatment-related deaths or significant toxicities. CRS was observed with varying severity but managed.	The efficacy was limited. No reduction in CD123 + cells was observed, and all patients progressed before day 28.	[149]
UniCAR-T with a targeting module (TM) for CD123	r/r AML	Flu/Cy	CRS was observed in most patients but was manageable, with no lasting myelosuppression or need for stem cell support.	The ORR was 53% in the r/r AML group and 75% in the MRD group. Some patients experienced durable remissions, with responses lasting up to five months.	[150]
CD7-CAR T-cell	r/r AML	Flu/Cy	Eight patients developed G 1–2 CRS, and 2 developed G 3 CRS.	Seven patients achieved CR (70% overall response rate). However, most patients relapsed due to CD7 loss. Two patients remained in a leukemia-free state at 752 and 315 days.	[54]
CLL-1-CAR T-cell	r/r AML	Flu/Cy	All eight children developed the CRS G 1–2.	Four patients achieved the MLFS and MRD negatively, one reached the MLFS with MRD positively, one achieved the CR with CRi but MRD positively, one experienced PR, and one remained in SD but had clearance of CLL1-positive AML blasts.	[151]
CLL-1-CAR T-cell	r/r AML	Flu/Cy	No differences were observed in the level of CRS between patients with and without EMDs.	Overall bone marrow CR rate of 65.00% in AML patients with EMDs and 81.48% in those without EMDs. In patients with EMDs, 55.00% achieved CR, with 10.00% achieving PR. Though not statistically significant, survival and remission duration were shorter in EMD patients	[152]
CLL-1-CAR T-cell	AML	Flu/Cy	CRS developed in all patients, with severe CRS occurring in 11 patients and severe ICANS in 2. These findings suggest that EASIX can predict severe toxicities and treatment outcomes, providing a valuable tool for patient management.	71.88% of patients achieved CR, and MRD became undetectable in 14 patients.	[153]
donor-derived CLL-1-CAR T-cell	r/r AML	Flu/Cy	Developed CRS G1. No GvHD was observed.	An 18-year-old male patient achieved CR on day 11 after CAR-T therapy. Leukocyte engraftment, complete donor chimerism, and platelet engraftment were observed after allo-HSCT	[154]
CLL-1-CAR T-cell and incorporated the apoptosis-inducing gene FKBP-caspase 9 (4SCAR system)	r/r AML	Flu/Cy	All patients experienced low-grade, manageable side effects, further supporting the potential of CAR T-cell therapy as an effective and safe treatment for targeting multiple myeloma stem cells.	Out of the four patients, three achieved CR with no detectable MRD, while the fourth patient survived for five months after treatment.	[155]
CD28/CD27 CLL-1 CAR T-cell and 4-1BB CAR T-cell	r/r AML	Flu/Cy	All patients developed G1 or 2 CRS, and one patient developed G2 ICANS	3 of 4 patients in the CD28/CD27 group and 2 of 3 in the 4-1BB patients achieved 75% CR and 65% CR, respectively.	[156]
dual CLL-1/CD33 CAR-T-cell	r/r AML	Flu/Cy	8 patients developed CRS (3 G1, 3 G2, 2 G3) and 4 patients developed ICANS.	Seven patients achieved remission with MRD negativity (78% overall response rate).	[157]
PD-1 silenced anti-CLL-1 CAR-T	r/r AML	Cy	Patient 1 received 1 × 10^7/kg of CLL-1 CAR-T-cells and developed Grade 1 CRS. Patient 2 received 5 × 10^6 cells/kg of CLL-1 CAR-T-cells and developed Grade 2 CRS.	Patient 1 achieved CR after undergoing allo-HSCT, which was maintained for 8 months. Patient 2 achieved morphological MRD- CRi on day 28	[62]
LeY-CAR T-cell	AML	Flu	No G3-G4 toxicities were observed.	Two patients achieved cytogenetic remission. One patient had blast count reduction but relapsed. One patient enrolled in another trial.	[158]
CD33-CAR T-cell	r/r AML	Not mentioned	Three patients (75%) experienced mild to moderate CRS, with one case having grade 4 CRS. Two patients developed mild neurotoxicity, and two also developed GvHD. All patients had grade 2–4 cytopenias and one developed sepsis.	Two patients (50%) achieved CR with CRi and were MRD-negative by day 30. The other two did not respond, though one had a PR after a second infusion. Two patients have remained disease-free for over two years, while one relapsed after one year.	[159]
CD33- CAR T-cell in 2 patients and CD123 CAR T-cell in one patient	r/r AML	FlA chemotherapy regimen and Flu/Cy	CRS G ≤ 3 And treated with tocilizumab. ICANS occurred in two patients, with G 1–2.	Two patients relapsed after 4 and 2 months, respectively. One patient remains in MRD-negative remission on day + 100.	[160]
CD33-CAR NK-92 cell	r/r AML	Case1 & 3: Ara-C + MTZ Case2: Flu + Ara-C + G-CSF	No significant adverse effects were observed at doses up to 5 × 109 (5 billion) cells per patient.	Case 1 (14-year-old girl): CR after chemotherapy & allo-HSCT. Relapsed with EMDAML after 15 months. Case 2 (24-year-old male): CR initially after chemotherapy but relapsed multiple times and died due to GvHD. Case 3 (49-year-old woman): CR after chemotherapy but relapsed with EMD skin lesions and later with 37.5% blasts in the bone marrow.	[161]
CD33-CAR NK cell	r/r AML	Flu and Cytoxan	71% of patients developed grade 1 CRS, and one developed grade 2 CRS.	The CD33-CAR NK cell was effective and safe. At day 28, 60% of patients achieved MRD-CR.	[162]
CD138-CAR T-cell	MM	Not mentioned.	No intolerable toxicity was observed during the treatment process.	Four of the five patients had stable disease for more than three months—advanced reduction plasma cell leukemia in myeloma cells in the peripheral blood in one patient.	[73]
GD2-CAR T-cell + inducible suicide caspase 9 (TRUCK)	GBM	Flu/Cy	Single and combined infusions of GD2-specific 4SCAR-T cells targeting GBM were safe and well tolerated, with no severe adverse events.	The infusion was intolerable and safe, with elevated levels of TNF-α and IL-6. All patients survived for more than six months. Four out of eight patients experienced PR. Three of them achieved PD, and one had SD.	[81]
EGFRvIII CAR T-cells + Pembrolizumab	GBM	Short-course radiation	No dose-limiting toxicity was observed.	While the combination was safe, the median progression-free survival and overall survival were relatively short. Alternative strategies may be needed to enhance the efficacy of this therapeutic approach.	[163]
Intrathecal bivalent EGFR and IL13Rα2- CAR T-cells	GBM	Radiotherapy/Temozolomide (RT/TMZ)	ICANS were observed in all patients, with one patient experiencing a dose-limiting toxicity.	The preliminary safety and bioactivity of the CAR T-cells were shown. Reductions in enhancement and tumor size were observed in all patients, but none met the criteria for objective radiographic response (ORR).	[164]
Intraventricular CARv3-TEAM-E T-cell	GBM	RT/TMZ	No significant side effects were observed.	All three patients showed dramatic tumor shrinkage within days of treatment. Two patients’ tumors eventually regrew, and one patient experienced durable tumor regression.	[165]
PSCA-CAR T-cell	Prostate	Flu/Cy	No dose-limiting toxicities were observed.	Showed preliminary safety and efficacy in mCRPC patients. Prostate-specific antigen declines were observed in 4 of 14 patients, along with radiographic improvements.	[166]
CD133-CAR T-cell	CRC + HCC+ pancreatic carcinomas	Nab-Paclitaxel and Cy	The primary toxicity is a decrease in hemoglobin/platelet (≤ grade 3) that is self-recovered within 1 week.	Of 23 patients, three achieved partial remission, and 14 achieved stable disease. The 3-month disease control rate was 65.2%, and the median progression-free survival was 5 months.	[135]
EpCAM-CAR T-cell	CRC + Gastric cancer	Not mentioned	Seven patients received the therapy, and all experienced hematologic toxicity, with some showing mild CRS:	Robust CAR-T-cell engraftment, cytokine elevation, and SD in four of five evaluable patients. One patient showed PD.	[112]
CD133-CAR T-cell	HCC	No Lymphodepletion therapy	The most common high-grade adverse event was hyperbilirubinemia.	Of 21 evaluable patients, 1 had a partial response, 14 had stable disease for 2 to 16.3 months, and six progressed after T-cell infusion.	[136]
EpCAM-CAR T-cell	solid tumors with lung metastasis	Cy	One patient with parotid gland cancer experienced grade 3 leukopenia.	One patient with colon cancer achieved PD. One patient with parotid gland cancer achieved a PR for 3 months and experienced tumor regression. Another patient with nasopharyngeal cancer maintained SD and achieved longer progression-free survival.	[167]
two-phase CAR T cocktail immunotherapy targeting EGFR and CD133	CCA	No Lymphodepletion therapy	The Patient experienced several obstacles, including vomiting, upper abdominal pain, chills, fever, elevated levels of IL-6 and TNF-α, and epidermal/endothelial damage.	A 52-year-old female patient achieved a PR lasting 8.5 months following the EGFR CAR T-cell and a PR lasting 4.5 months following the CD133 CAR T-cell.	[168]

Abbreviations: Flu: Fludarabine, Cy: Cyclophosphamide, Ara-C: Cytarabine, MTZ: Mitoxantrone, CRC: Colorectal cancer, CCA: Cholangiocarcinoma, PFS: Progression-free survival, OS: Overall survival, EMD: Extramedullary disease, MRD: Minimal residual disease, MLFS: Morphologic leukemia-free state, PR: Partial remission, SD: stable disease, CR: completer remission, CRi: Complete remission with incomplete hematologic recovery, EASIX: Endothelial activation and stress index, SCT: Allogeneic stem cell transplantation, HCC: Hepatocellular carcinoma, PD: Progression disease, CAR: Chimeric antigen receptor, CRS: Cytokine release syndrome, GBM: Glioblastoma, TNF: Tumor necrosis factor, PFS: Progression-free survival, IL: Interleukin, RT/TMZ: Radiotherapy/Temozolomide, AML: Acute myeloid leukemia, ICANS: Immune effector cell-associated neurotoxicity syndrome, GvHD: Graft versus host disease

## Anti-CSC CAR-based treatment challenges

Although treatment with CAR T cell has created revolutionary breakthroughs in cancer treatment, many barriers to using anti-CSC CAR T cell therapy must be addressed (Table 2). These challenges include CAR T-cell-associated toxicities and factors that impair the treatment’s effectiveness, such as tumor heterogeneity, an immune-suppressive TME, and limited CAR T-cell trafficking. The primary toxicities linked to CAR T-cell therapy are CRS, immune effector cell-associated neurotoxicity syndrome (ICANS), and on-target off-tumor toxicity. CRS is characterized by a surge of inflammatory mediators, including cytokines and chemokines, produced by the infused CAR T-cells and monocytes, macrophages, and dendritic cells. Key cytokines involved in CRS include IFN-γ, TNF-α, GM-CSF, IL-1, IL-2, IL-8, IL-10, and IL-6 [169]. These factors contribute to a range of CRS symptoms, such as fever, fatigue, muscle pain, joint pain, chills, anorexia, low blood pressure, rapid heart rate, shortness of breath, hypoxia, arrhythmia, capillary leak, blood clotting issues, respiratory failure, shock, and organ dysfunction. Several elements play a role in the development of CRS, including a high tumor burden, the large number of CAR T-cells administered, a significant peak in CAR T-cell expansion, pre-existing thrombocytopenia, endothelial activation before treatment, and the presence of a CD28 costimulatory domain. Also, lymphodepletion makes CAR-T cells more prone to activation and expansion, which can cause CRS. Therefore, these risk factors should be carefully considered before administering CAR-based therapies [170]. Cardiac side effects are linked to the severity of CRS, with IL-6 playing a crucial role in this process. As a key cytokine involved in CRS development, IL-6 attracts additional T-cells, increasing cytokine production and exacerbating CRS’s severity. Furthermore, IL-6 activates prostaglandins, leading to tachycardia, low blood pressure, and multi-organ failure. Consequently, the concentration of IL-6 is directly associated with the intensity of cardiac side effects [171]. Risk factors for cardiac side effects in CAR T-cell therapy include having more than 25% blasts in the bone marrow before treatment, experiencing Grade III or higher CRS, a history of diastolic heart dysfunction, advanced age, low ejection fraction before CAR T-cell infusion, coronary artery disease, elevated lipid levels, aortic stenosis, high baseline creatinine, and increased troponin levels. Cardiac complications may present as hypotension, left ventricular dysfunction, and shock. Critical interventions for managing these toxicities involve supportive care, IL-6 inhibitors, and corticosteroids [171, 172]. ICANS is neurotoxicity related to CAR T cell therapy that has no obvious pathophysiology. However, it is assumed that ICANS may contribute to blood-brain barrier (BBB) disruption following endothelial activation and effusion of cytokines to the central nervous system passively [173]. In addition to CRS risk factors, other factors contribute to ICANS, including experiencing neurological conditions, high lactate dehydrogenase (LDH) levels, thrombocytopenia and endothelial stimulation before CAR T cell treatment, and high ferritin concentration within 72 h after CAR T cell administration [174]. Symptoms of ICANS are diverse and may start with attention deficit and confusion, aphasia, and handwriting changes, and can progress to impaired consciousness, coma, seizures, motor weakness, and fatal neurotoxicity due to cerebral edema in higher grades [175]. One significant toxicity related to CAR T-cell therapy, particularly in anti-CSC CAR T-cell treatments, is On-target Off-tumor toxicity. This occurs because certain TAAs, although primarily expressed in CSCs, are also in low amounts in normal tissue cells. When CAR T-cells interact with these normal cells, it can damage healthy tissues, causing On-target Off-tumor toxicity. For instance, CD133, a commonly cited CSC marker, is also expressed in normal neural stem cells, potentially leading to unintended destruction by anti-CD133 CAR T cells [101]. Other reasons for On-target off-tumor toxicity may be the secretion of inflammatory or cytotoxic mediators and/or upregulation of T cell surface molecules to induce target apoptosis [176]. Other toxicities associated with CAR T-cell therapy include infusion reactions, tumor lysis syndrome, anaphylaxis, and immunogenicity. Additionally, patients may experience B-cell aplasia and hypogammaglobulinemia, increased susceptibility to infections, hemophagocytic lymphohistiocytosis/macrophage activation syndrome, as well as genotoxicity and the potential development of secondary malignancies [5].

The cellular diversity of cancer, especially in solid tumors, which includes blood vessel cells, differentiated cancer cells, CSCs, and immune cells, is one of the primary causes of treatment failures. These cells simultaneously impact several cellular processes essential to the development of cancer. “Tumor heterogeneity” often refers to intra-tumor heterogeneity, variations among cells inside individual tumors, and inter-tumor heterogeneity, or variances seen at the population level that directly impact treatment response and prognosis. Also, the phenotype plasticity of cancer cells during the treatment is that cancer cells that are sensitive to treatment polarize toward drug-resistant phenotypes. In the clinical application, tumor heterogeneity and plasticity complicate therapeutic targeting. For instance, via converting low-expression CD-44 phenotype as non-CSCs to high-expression CD-44 phenotype as CSCs, new CSCs arise that contain new markers, which lead to a reduction in treatment effectiveness [11]. Furthermore, mutations in cancer cells or lineage switching could enable tumors to evade recognition by CARs [177].

Another mechanism that could cause treatment failure is antigen escape. This escape could occur in various ways, including genetic alterations, epigenetic modifications, immune editing, antigen shedding, and clonal selection [178]. After eliminating CSCs, another mechanism could arise: the dedifferentiation of non-CSCs to CSCs [9]. This highlights the importance of targeting both CSCs and non-CSCs to ensure complete tumor elimination and prevent recurrence. This bidirectional shifting between CSCs and non-CSCs can addressed by designing multi-targeting CAR T cells and combination therapy approaches. Additionally, CAR T-cell therapy faces challenges with low infiltration into the TME due to physical barriers and the downregulation of molecules like intercellular adhesion molecule-1 (ICAM-1) and vascular cell adhesion protein-1 (VCAM-1). Tumor-derived factors, such as VEGF-A, can suppress the expression of these adhesion molecules on endothelial cells lining blood vessels, which are essential for the binding and migration of immune cells, including CAR T-cells, from the bloodstream into the tumor. When these molecules are downregulated, CAR T-cells face difficulty crossing the endothelial barrier and reaching the tumor tissue [179]. Diverse tumor-related chemokines such as CCL2 and CCL17/22 and various immunosuppressive tumor cells such as regulatory T-cells, MDSCs, and cancer-associated fibroblasts (CAFs), and their derivatives are the other reasons accompanied by inhibition of immune cells, like T-cells, infiltration into the tumor niche [179]. The persistence of CAR T-cells is the duration of T-cell exhaustion and dilapidation. The main factor that leads to CAR T cell exhaustion is immunosuppressive TME. The TME is a complex 3D network architecture with various compartments, including cancer, immune, endothelial, CAFs, and ECM. Each of these compartments has a bidirectional relationship with each other. The immunosuppressive factors in the tumor niche and its circumference include metabolic and physical barriers, immunosuppressive molecules, immunosuppressive cells, and their derivations [180]. Also, CSCs have various roles in the production of this microenvironment. We could mention T regulatory cells, TAMs, tumor-associated neutrophils (TANs), MDSCs, and CAFs as immunosuppressive cells. Also, factors and pathways like IL-4, IL-13, IL-6/STAT3, PD-1, LAG3, CTLA-4, Tim3/Galectin-9 signaling pathway, CXCL9, CXCL10, Hypoxia /HIF1α, contribute to constructing TME and barriers. Any of these factors or cells have a certain role(s) in constructing immunosuppressive TME. Also, most have a strong relationship with CSCs and their properties, such as metastasis, angiogenesis, immune escape, drug resistance, and tumor invasion [181]. These are the main reasons for impairment in immune cell persistence, infiltration, and function in tumor niches. The long-term persistence of CAR T-cells in the body could be crucial in preventing cancer relapse. Also, it is known as a response rate factor in this therapeutic manner. CAR T cell persistence is influenced by immune responses to CAR, transgene expression stability, and CAR T cell adaptability inside the body [101, 182].

The Off-the-shelf CAR-based therapy is about the time and process of construction, cost, and fast accessibility of these cells. Also, due to multiple prior treatments that the patients who participate in clinical trials of this therapeutic approach usually receive, autologous immune cells are harmfully affected by this treatment, which could be another issue in the off-the-shelf field. An overcome approach to these subjects is manufacturing allogeneic CAR T-cells from healthy donors that could decrease the cost of manufacturing, give us a high number of immune cells, and be prepared in advance. However, this approach faces some disadvantages, such as Immune rejection, GvHD, CAR T cell fratricide, or suicide [183] (See Table 3).

**Table 3:** Mentions several adverse effects of CAR-based therapies, including CRS, ICANS, on-target off-tumor toxicity, tumor heterogeneity, antigen escape, trafficking and infiltration, persistence, and suppressive tumor microenvironment. Each of these is explained separately, as are their solutions

Challenge	Description	Management(s)/overcoming approach(es)
CRS	CRS typically begins with fever and constitutional symptoms like rigors, malaise, and anorexia. The fever is often high-grade and can persist for several days. In more severe cases, CRS can progress to include other signs of a systemic inflammatory response, such as hypoxia, hypotension, and organ dysfunction [170].	1. Supportive treatment 2. Corticosteroids 3. Monoclonal antibodies against inflammatory mediators 4. Addressing risk factors [15]
ICANS	ICANS typically presents as toxic encephalopathy, starting with symptoms such as word-finding difficulties, confusion, dysphasia, aphasia, impaired fine motor skills, and tiredness. In more severe cases, it can progress to include seizures, motor weakness, cerebral edema, and even coma [170].	1. Supportive treatment 2. Steroids such as dexamethasone and methylprednisolone 3. Addressing risk factors [170]
On-target off-tumor toxicity	Interaction between CAR T-cells and TAAs, expressed by normal cells in low amounts, causes damage to normal tissues [101). Another hypothesis for this side effect is the secretion of inflammatory or cytotoxic mediators and/or upregulation of T cell surface molecules to induce target apoptosis [174].	1. Utilizing dual-targeted CAR-T-cells 2. Intra-tumoral injection of CAR-T-cells 3. Using controllable CAR T-cells with a safety switch 4. CAR T cell with inhibitory CAR [15]
Tumor heterogeneity	This refers to tumor heterogeneity, which describes the diversity of cellular populations either between tumors of the same type in different patients (inter-tumoral heterogeneity) or within a single tumor (intra-tumoral heterogeneity) [178].	1. Eradication of both CSCs and non-CSCs for the elimination of whole cancer and prevent recurrence 2. combination therapy 3. Utilize dual/multi-target CAR T-cells 4. Design BiTEs secreting CAR T-cells to summon bystander T-cells to target another TAA [15]
Antigen escape	A well-established tumor-intrinsic mechanism of immunotherapy resistance. The therapeutically enhanced T cell immunity exerts selective pressure on the tumor, which enables the outgrowth of subclones with low or absent target antigens, resulting in tumor immune editing and alterations in the antigenic landscape [178].	1. Combination therapy 2. Utilize dual/multi-target CAR T-cells 3. Design BiTEs secreting CAR T-cells to summon bystander T-cells to target another TAA [15]
Trafficking and infiltration	The dense fibrotic matrix, along with tumor-related chemokines like CCL2 and CCL17/22, and various immunosuppressive tumor cells, such as regulatory T-cells, MDSCs, and CAFs and their derivatives, create physical and immune barriers that hinder the penetration of CAR T-cells into the tumor microenvironment. This impedes the effectiveness of CAR T cell therapies in solid tumors [179].	1. Combination therapy with CAR cells and radio/chemotherapy 2. Local delivery of immune cells 3. Design specific CAR cells that express attractive chemokines and chemokine receptors 4. Altering cancer signaling pathways, such as raising the expression of CCR4, CCR2b, or CXCR3 ligands 5. Construct CAR T-cells that express ECM-modifying heparinase enzymes. 6. Use fibroblast activation protein (FAP)-directed CAR-T-cells for preventing matrix production and angiogenesis of cancer [87, 101, 15].
Persistence	The persistence of CAR T-cells refers to how long they remain active in the body before experiencing exhaustion and degradation. The stability of transgene expression, immune responses against the CAR construct, and the adaptation of CAR T-cells to the body all influence their long-term persistence. The sustained presence of CAR T-cells is crucial for preventing cancer relapse and is associated with improved treatment response rates [101, 182].	1. Incorporating certain types of costimulatory domains like 4-1BB, ICOS, OX40 or CD27 in CAR structure 2. Combination therapy with ICIs 3. Utilize cytokine-secreting CAR T-cells such as those that secrete IL-12, IL-18, IL-7, IL-15, and IL-21 4. Design JAK/STAT signaling CAR T-cells [15]
Suppressive tumor microenvironment	In the TME, various tumor-infiltrating cells, including TAMs, MDSCs, T-regs, and CAFs, play critical roles in immune suppression. These cells produce growth factors, cytokines, and chemokines that promote tumor growth and contribute to establishing an immunosuppressive microenvironment, hindering the effectiveness of immune therapies such as CAR T-cell therapy [180].	1. Modification and neutralizing its factors 2. Engineering CAR T-cells with the capability of expressing IL-12, IL-18, IL-7, IL-15, and IL-21 3. Combination therapy with ICIs [15]

Abbreviations: CRS: Cytokine Release Syndrome, ICANS: Immune effector cell-associated neurotoxicity syndrome, CSC: Cancer stem cell, TAM: Tumor-associated macrophage, MDSC: Myeloid-derived suppressor cell, CAF: Cancer-associated fibroblast, TME: Tumor microenvironment, ICI: Immune checkpoint inhibitor, BiTEs: Bispecific T cell engager, TAA: Tumor-associated antigen CAR: Chimeric antigen receptor

## Tide turned in the anti-CSC CAR against tumors. The arts help us prevail on the stage

### Enhancing CAR structure

**Fig. 3:**
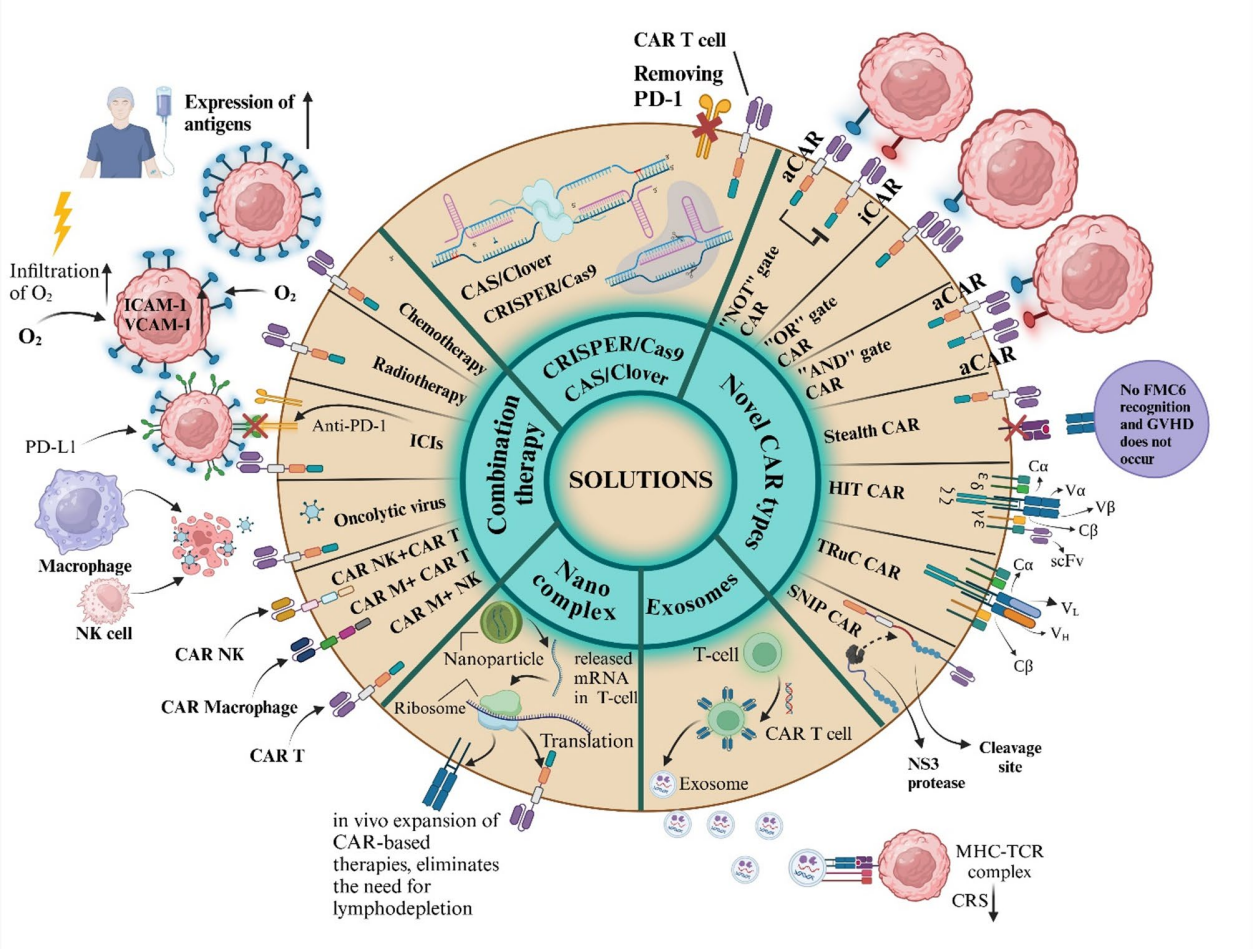
The anti-CSC CAR T-cells improvement methods. The anti-CSC CAR T-cells have several challenges in their progression pathways, requiring some solution methods. In different surveys, many solutions have been introduced, some of the important ones shown in the above figure, but other ones like artificial intelligence combination, novel CAR generations, and other genetic modification pathways are other solution approaches that can be used in this manner. Attention to these solution pathways is necessary for future investigations to achieve the best outcomes in tumor elimination. Abbreviations: ICIs; immune checkpoint inhibitors, PD-1: programmed-cell death 1, ICAM-1: Intercellular adhesion molecule-1, VCAM-1: Vascular cell adhesion protein-1, HIT CAR: HLA-independent T cell receptors CAR, TRuC CAR: TCR fusion construct CAR, SNIP: signal neutralization by inhibitable protease

As mentioned above, the anti-CSC CAR has various challenges in the treatment manner and failed in the cancer treatment, especially in solid tumors and r/r one’s elimination. This setting has mentioned some methods to help us overcome the challenges (Fig. 3). These arts can be categorized in some manner, such as improvement of the CAR structure, combination therapy, promotion of the manufacturing process, and preparation of the best field for CAR action.

CAR structure can be improved by using novel CAR types/ generations. For instance, for targeted action and prevention of toxicities, CAR-based therapies are functionally classified into “AND”, “OR”, “NOT”, and “IF-THEN” logic-gated CAR T-cells. Each type is further categorized into structural subtypes. Additionally, other platforms of CAR-based therapies focus on enhancing persistence, preventing exhaustion, and avoiding immune system evasion. “AND” logic-gated CARs require approaches simultaneously targeting multiple antigens. For instance, bispecific CARs are designed to target two antigens, like dual CARs concomitantly. Dual CARs have two separate CARs on the same T cell, each operating independently and only being stimulated when two antigens have been targeted simultaneously. Another “AND” logic-gated CAR is called synNotch CAR, which operates based on a synthetic Notch receptor. This receptor releases a transcription factor upon binding to antigen A, which stimulates the expression of a second CAR to eliminate antigen B. Due to its function, it can be categorized as an “IF-THEN” logic-gated CAR. Targeting two markers simultaneously leads to beneficial outcomes in overcoming on-target off-tumor toxicities, similar to “NOT” logic-gated CARs [184]. “NOT” logic-gated CARs operate based on inhibition, sending inhibitory signals to prevent the CARs’ function in the presence of specific antigens, thereby preventing toxicities to normal tissues, which is designed with two receptors: one for activating CAR and one for inhibiting CAR. This CAR allows targeting the stem cell marker while sparing healthy tissues. During the elimination of the targeted markers, the inhibiting CAR prevents damage to healthy tissues that express the targeted markers. “OR” logic-gated CARs are an approach that operates in a broader application, as they stimulate bispecific CARs by targeting either one of the antigens, like tandem CARs, which have two ScFv arranged in one CAR to recognize two antigens and can stimulate either target two antigens or one antigen. Therefore, it is not necessary to target two antigens simultaneously. However, “OR” logic-gated CARs are unsuitable for targeting TAAs also expressed on normal tissues due to the susceptibility to off-tumor on-target toxicities. Therefore, careful selection of antigens for targeting is essential [185].

Indeed, certain CAR-based constructs are designed to enhance persistence and improve the effectiveness of CAR T-cell therapy. One such approach is the development of Signal Neutralization by Inhibitable Protease (SNIP) CARs. These CARs are engineered to remain inactive until exposed to a specific drug, which activates the CAR and allows it to function. This drug-controllable mechanism helps prevent premature activation of CAR T-cells, potentially reducing unwanted side effects while ensuring that the cells remain effective when needed. This approach could be beneficial in regulating CAR T-cell activity within the complex TME [184]. Another innovative CAR design is the stealth CAR, which addresses challenges related to immune system rejection and GvHD. It benefits off-the-shelf applications, similar to the CAR NK cell platform. In a recent study, viral inhibitors of transporter associated with antigen processing (TAPi) were combined with a transgene encoding shRNA targeting class II MHC transactivator (CIITA). This combination reduced the expression of both MHCI and MHCII, thereby minimizing the likelihood of immune rejection. Despite this modification, the efficacy of the novel stealth CAR T-cells was retained, demonstrating promising anti-tumor effects both in vitro and in vivo. This strategy offers a potential advancement for broader use of CAR T-cells with reduced risks of immune-mediated complications [186]. Another CAR type is HLA-independent T cell receptors (HIT CAR), designed to exhibit superior anti-tumor activity with high sensitivity. HIT CARs can eliminate tumors without the constraints of HLA presentation, broadening the range of targetable cancers. In this CAR, a TCR is modified and engineered by replacing the variable domain of the endogenous TCR with an scFv through gene editing at the TRAC locus. This allows the recognition of TAAs in an HLA-independent manner [187]. Also, the TCR fusion construct (TRuC) CAR is another structure where the entire TCR complex is fused directly to an ScFv, operating without co-stimulatory signals like HIT CAR. This CAR can also stimulate and operate against specific markers in an HLA-independent manner, providing the potential for extensive applications despite secreting low levels of cytokines [184]. A study utilized a dual peptide construct (EV) to recognize and bind CSC antigens. To evaluate the functionality of this novel CAR T cell, the EV construct was incorporated into the CAR T cell in place of the conventional ScFv. This modification aimed to target CSC antigens with greater specificity. The EV construct binds to CSCs through N-cadherin, which is overexpressed in GSCs relative to normal tissues and differentiated glioma cells. This overexpression facilitates the targeted binding of the EV construct to CSCs, thereby enhancing its feasibility as a therapeutic approach. Moreover, the engineered CAR T-cells exhibited significant cytotoxicity and potential for targeting CSC antigens [188]. As a result, various types of CARs have been designed in recent years to improve their efficacy in treating hematological and solid tumors and overcome poor persistence and off-tumor on-target toxicities.

### Combination therapy

On the other hand, recent data demonstrate that CAR monotherapy cannot effectively impact cancer cells. Although some data have shown the efficacy of CAR monotherapy, combination therapy can help overcome some of its limitations. Combination immunotherapy has been a significant development in cancer therapy for many years, highlighting the need to eliminate CSCs within tumors. One combination therapy method combines each immune cell with another in the CAR platform or without it. One of these examples of CSC markers is CAR T-cells with CAR NK cells. Although no clinical trials have yet combined CAR T-cells and CAR NK cells specifically against CSCs, some studies suggest that these engineered cell types may function similarly when targeting cancer cells, including CSCs. For example, a study by Li et al. explored the use of CAR T-cells and CAR NK cells to target CD19-positive tumors. The results demonstrated that this combination therapy resulted in a lower incidence of CRS, better persistence of CAR NK cells in vivo, and greater overall therapeutic efficacy. Moreover, the combination significantly improved xenograft survival and prevented tumor relapse for several months. These findings highlight the potential advantages of CAR T-cells and CAR NK cells in cancer therapy [189]. Indeed, the CAR T and CAR M combination had significant synergistic results in tumor killing due to their supplementary roles like high homing and infiltration of CAR M in addition to high cytotoxicity and easy production of CAR T-cells [190]. As a hypothesis, combining other immune cells in the CAR platform, like CAR NK and CAR M, can be used in future studies due to their complementary roles in the immune system.

Furthermore, the other SoC methods can be combined with CAR-based treatment to achieve better outcomes. These methods are chemotherapy, radiotherapy, photothermal therapy, immune checkpoint inhibitors (ICIs), and oncolytic viruses that help us to overcome CAR-armored immune cell gaps. For many years, chemotherapy has been the standard cancer treatment, but its efficacy against CSCs is limited due to their unique ability to cause relapse even after an initial response [191]. Combining chemotherapy with CAR-based therapies can enhance treatment effectiveness by addressing challenges such as immunosuppressive cells, antigen loss, and recurrence [192]. Similarly, radiotherapy can complement CAR T cell therapy by reducing challenges like antigen loss, poor CAR infiltration, and angiogenesis. Radiotherapy enhances immune responses, promotes CAR T cell expansion, and aids tumor infiltration, potentially reducing side effects. For example, it can normalize tumor vasculature, improve oxygenation, and upregulate ICAM-1 and VCAM-1, facilitating CAR cell entry into the tumor. Additionally, radiotherapy induces inflammation via pathways like IFN-γ and STING, promoting the recruitment of effector T-cells through chemokines like CXCR3, CXCL9, CXCL10, and CXCL 11 [179]. A synergistic effect has also been observed between CAR NK cells and radiotherapy, which improves CAR NK infiltration, stimulates the release of IFN-γ and TNF-α, and attracts CAR NK cells to tumors via chemokine release [193]. However, radiotherapy has downsides, such as ATP release from cancer cells converting to adenosine, which impairs T-cell activity via A2a receptors [179]. This can be mitigated by removing A2a receptors using CRISPR-Cas9. Radiotherapy also induces TGF-β in the TME, promoting immunosuppression and hindering CAR-based therapies [179]. Further research is needed to find the optimal radiotherapy dose for combined CAR-based therapies across different cancers. Following radiotherapy, another promising strategy to enhance CAR-based therapies is the combination with ICIs. Radiotherapy can boost CAR T cell infiltration and immune activation, and ICIs further amplify this effect by targeting immune checkpoints expressed by cancer cells, which hinder immune cell function. Although ICIs alone show limited response rates in many cancers, combining them with CAR therapies has shown more robust outcomes, making it a preferred option over CAR monotherapies [194]. Moreover, various studies utilized novel combination therapy, like combining CAR T-cells with metabolic regulators, anti-VEGF therapy, etc. For instance, one study explored the impact of metabolic conditions on CAR T cell function in glioma tumors expressing the EGFRvIII mutation (SB28-EGFRvIII). These tumors, known for their low oxygen levels, caused impairment in CAR T-cells due to reduced mitochondrial ATP production. To address this, the study pre-treated mice with metabolic regulators, such as Rapamycin (an mTOR inhibitor) and Metformin (an AMPK activator), to enhance CAR T cell function. The pre-treatment activated PPAR-gamma coactivator 1α (PGC-1α), which improved mitochondrial spare respiratory capacity in CAR T-cells. As a result, the treatment led to better CAR T cell infiltration into the tumor and a reduction in the Ly6c + CD11b + monocytic myeloid-derived suppressor cells, enhancing CAR T cell therapy [195]. Another study used anti-VEGF therapy in combination with EGFRvIII-CAR T-cells. The anti-VEGF drug enhanced the infiltration and distribution of CAR T-cells and improved mice survival compared to EGFRvIII-CAR T-cells alone [196].

Critical signaling pathways such as Notch and Wnt/β-catenin play a key role in maintaining CSC stemness, participating in tumor progression, and treatment resistance. Targeting these pathways can enhance CAR T cell therapy efficacy by disrupting CSC self-renewal and differentiation capabilities [197]. Notch inhibitors, such as γ-secretase inhibitors (GSIs), have been explored to suppress CSC survival and sensitize tumors to immunotherapy. Similarly, Wnt/β-catenin pathway inhibitors, including small-molecule inhibitors like PRI-724 and LGK974, can impair CSC proliferation and reduce immune evasion [198, 199]. Thus, another promising approach involves combining CAR T cells with signaling pathway inhibitors in CSCs, where Notch or Wnt suppression weakens CSCs. Also, CSCs exhibit unique metabolic adaptations that support their survival, self-renewal, and resistance to therapy. Unlike non-CSCs, which primarily rely on glycolysis, CSCs display metabolic plasticity, switching between glycolysis and oxidative phosphorylation (OXPHOS) based on environmental conditions [197, 200]. This adaptability allows CSCs to evade immune attacks and sustain tumor growth. Targeting CSC metabolism presents a promising strategy to enhance CAR T cell therapy by making these cells more vulnerable to immune elimination [200].

### Facilitating the CAR T cell production

A key strategy in CAR-based treatments is optimizing the CAR production and activity process. Exosomes, which carry molecules like microRNA, mRNA, DNA, and proteins, facilitate communication between cells locally or by traveling through the bloodstream [201]. CAR T cell-derived exosomes hold promise for overcoming challenges like CD8 + T cell exhaustion and resistance to PD-1 blockade therapies [202]. These exosomes can deliver tumor-targeting receptors and cytokines to enhance antitumor effects while avoiding toxicities such as CRS, as they do not expand uncontrollably. Functionally similar to CAR T-cells, CAR T cell-derived exosomes can interact with tumor antigens, creating a TCR-MHC complex to deliver cytotoxic molecules that kill cancer cells [201]. They are also less immunogenic, allowing for off-the-shelf applications [203]. Additionally, CAR T cell-derived exosomes can cross biological barriers, penetrate solid tumors, reduce neurotoxicity, and counteract PD-L1-mediated immunosuppression [201]. Consequently, given the beneficial outcomes of CAR T exosomes, they can be used as a novel alternative approach. Several pieces of evidence suggested novel techniques that are innate and adaptive immune cell-derived exosomes, including NK cells, M1 macrophages, neutrophils, B cells, CD4 + cells, and CD8 + cells [204]. There is mounting evidence demonstrating the efficacy of exosomes derived from innate immune cells. For instance, NK cell-derived exosomes and M1 macrophage-derived exosomes have exhibited remarkable cytotoxic effects against metastatic and hematological cancers. One study indicated that exosomes from NK-92 cells stimulated by IL-15 and IL-21 showed increased cytolytic activity against lung and cervical cancers [205]. Thus, exosomes from the innate immune system play important roles and benefit the immune response against malignant tumors [204]. These exosomes can be engineered to interact with other immune cells and enhance the immune system’s ability to eliminate tumors, highlighting the need for further investigations against CSCs.

Another approach to promote CAR activation is based on a gene-editing tool. CAR T cell exhaustion is a severe challenge that needs to be overcome. The primary reason for CAR T cell exhaustion is immune checkpoints, such as PD-1 and CTL-4, which are crucial for inhibiting T cell activation. Thus, an innovation was needed for the novel design of CAR T-cells to eliminate the immune checkpoint selectively [206]. This novel design leverages the gene-editing technology known as the clustered regularly interspaced short palindromic repeats and CRISPR-associated protein 9 (CRISPR/Cas9) system [207].

Utilizing the CRISPR/Cas9 system to remove PD-1 promotes CAR T-cells’ persistence and anti-tumor response. Various CSC markers have been targeted, demonstrating the potential effect of the anti-tumor activity of CRISPR/Cas 9 technology. For instance, certain studies employed the CRISPER/Cas9-mediated knockout of PD-1 and other ICIs (TRACK, B2M, and DGK) in CAR T-cells targeting EGFRvIII in glioblastoma, resulting in improved anti-tumor responses [208, 209]. Furthermore, due to the advantages of CRISPR/Cas9 editing technology, CAR T-cells achieve higher persistence than conventional CAR T-cells. This reduces the required dose of CAR T cell infusion and the treatment course, consequently lowering the cost of CAR T cell manufacturing [206]. As a result, the CRISPR/Cas9 system has shown significant promise in addressing various challenges, playing a pivotal role in optimizing CAR T-cell therapy. However, further research is necessary to explore advanced CRISPR/Cas9 technologies, such as Cas13, Cas14, Cas12a, and the nickase Cas9 (nCas9). These systems could offer improved efficiency and precision in genetic modifications, enhancing the effectiveness of CAR T-cell therapies [206, 210]. Due to the risk of off-target mutations with CRISPR/Cas9, a novel gene editing tool was developed to address this issue. Cas-CLOVER is a novel editing tool that utilizes two inactive Cas9 (dCas9) proteins, each fused with the Clo051 nuclease domain. When two separate guide RNAs (sgRNAs) bind to their specific DNA sequences, the Clo051 enzyme is activated and induces a precise cut at the target DNA. This dual sgRNA requirement enhances the accuracy of Cas-CLOVER, reducing the risk of off-target mutations. As a result, the dual-guided strategy in Cas-CLOVER promotes greater fidelity compared to CRISPR/Cas9. This novel technology reduces the risk of off-target mutations. Unlike CRISPR/Cas9 and other gene editing tools that require activating T-cells, it allows for gene editing in resting T-cells. This innovative tool will enable researchers and practitioners to preserve stem cell memory T-cells (TSCM). Furthermore, Cas-CLOVER offers the potential to produce off-the-shelf CAR T-cells by suppressing T cell receptor beta constant (TRBC) or T cell receptor alpha constant (TRAC) and B2M, thereby removing the endogenous T cell receptor and MHC class I molecules, respectively [211]. Additional investigations are required to ensure further the CAS/CLOVER system’s safety and feasibility. Two Phase I clinical trials are currently being conducted to evaluate the effects of CAS/CLOVER-mediated knockout of the TRBC and B2M genes in CAR T-cells targeting MUC1C-positive tumors in multiple myeloma (NCT04960579, NCT05239143).

Another approach that promotes the activation of CAR-based therapies and improves their production, which is still under investigation, is in vivo production. If this process is successfully carried out, it could revolutionize cancer immunotherapy. In ex-vivo production, multiple steps are required, such as leukapheresis, T cell transduction, CAR T cell expansion, lymphodepletion, and infusion of CAR T cells into the body [15]. Each step presents challenges, including manufacturing failure, technical errors, and high costs. Thus, the ex-vivo process demands significant caution. In vivo production offers a potential solution to address these issues. Of in vivo output, vectors are infused to transduce T-cells with gene material encoding the CAR structure rather than isolating T-cells. This approach simplifies CAR-based therapy production and eliminates the need for lymphodepletion and bridge therapy. As a result, in vivo, production offers numerous patient benefits, including streamlining the production process, reducing costs, avoiding delays in patient preparation, and eliminating the need for lymphodepletion [212].

### Nano complexes

Several solutions are presented to overcome the limitation of CAR-based therapies manufacturing and their functions. One suitable solution is to integrate nanomaterials into the structure of CAR-based therapies. The type of nano complex can be utilized in CAR-based therapies, such as CAR T-cells, CAR M, and CAR NK cells. For instance, A study demonstrated that delivering plasmid DNA encoding CAR with interferon-γ with the mannose-conjugated polyethyleneimine (MPEI) nanocarriers, which target macrophages, induce the macrophages to convert into M1 macrophages, leading to potential direct attack, as well inhibition of solid tumors in living organism [213]. Several types of nanomaterials can be employed for T/NK cell expansion and enhancement, functioning as artificial antigen-presenting cells (aAPCs) [214]. These nanomaterials, such as magnetic nanomaterials, polymer nanoparticles, carbon nanomaterials, lipid bilayers, and DNA origami, improve T/NK cell activation by mimicking the properties of natural APCs. Additionally, nanomaterials like cationic polymers, liposomal nanoparticles, lipid-polymer hybrid nanoparticles, and those optimized for electroporation are used for efficient gene delivery, including CAR genes to T/NK cells [214, 215]. Researchers have also developed innovative nanoparticle backpacks equipped with thiol-reactive groups, which can release specific proteins upon antigen induction, enhancing the effectiveness of CAR-based therapies. These backpacks can be engineered to act as antibodies, secrete cytokines, or release small molecule inhibitors, providing a controlled and potent therapeutic response [215].

### How AI could improve Anti-CSC CAR T cell therapy

Recent advances in computational processing and deep learning algorithms have made AI more prominent in cancer patient management. The AI could assist in various aspects such as tumor diagnosis, tumor segmentation, TME evaluation, drug design, and response prediction [216]. In this part, we discuss how AI methods can improve the clinical results of CAR T cell therapy. First, as mentioned above, due to different TME intrinsic and extrinsic characteristics among patients, the response to the same CAR T cell product varies. Therefore, it is appropriate to analyze TME and other characteristics of patients, such as age, race, and sex, before receiving CAR T-cells. This could be addressed by applying machine learning-based methods like radiomics, which give us important information about cellular architecture and biological processes in TME without invasive methods like biopsy [217]. As a result, AI could guide clinicians in detecting patients who benefit most from treatment. Second, utilizing the optimal CAR structure to produce CAR T-cells with high cytotoxicity and stemness is crucial. Through machine learning and an arrayed screen of several hundred receptors, we could identify design principles associated with cell destiny and understand the fundamental elements of the signaling motif language. The objective of creating receptors with the most efficient signaling domains is to design CAR T-cells with high cytotoxicity against cancer cells and long persistence [218]. Third, patient follow-up and response assessment are essential because some patients may experience relapse or recurrence. Also, anti-cancer drugs that are administrated to patients, especially immunotherapy drugs, can augment inflammatory response and mimic cancer progression in conventional MRI. This condition (pseudo-progression versus true-progression) makes accurate evaluation of patients difficult. To distinguish between pseudo-progression and true-progression more accurately, AI-based methods such as radiomics, radio-genomics, and radio-pathomics have been employed in various studies [219]. For instance, researchers employed three machine learning algorithms in a survey to predict progression within 6 months after CAR T cell therapy in R/R B-NHL patients using radiomic features derived from contrast-enhanced CT. No notable differences were observed between the areas under the curves (AUCs) of the combined and radiomic models for all machine-learning algorithms in the test set. Radiomics and combined models constructed with multi-lesion-based radiomic features demonstrated higher predictive performances than those utilizing the most extensive lesion-based radiomic features [220].

## Conclusion and future prospectives

Using CAR T-cells to target the CSC population is a promising approach to eliminating cancer as it shows notable clinical results in various studies. However, more clinical studies are required to establish anti-CSC CAR T cell therapy’s clinical efficacy and possible toxicities. Also, using other candidates like CAR NK, CAR NKT, and CAR macrophages instead or in combination with CAR T to target CSCs may lead to better clinical results. Furthermore, due to the significant progress of AI in recent years, it could help us to reach optimal clinical results in anti-CSC CAR T cell therapy by designing the most potent CAR T cell drug and selecting patients who benefit most from treatment.

**Figure:**
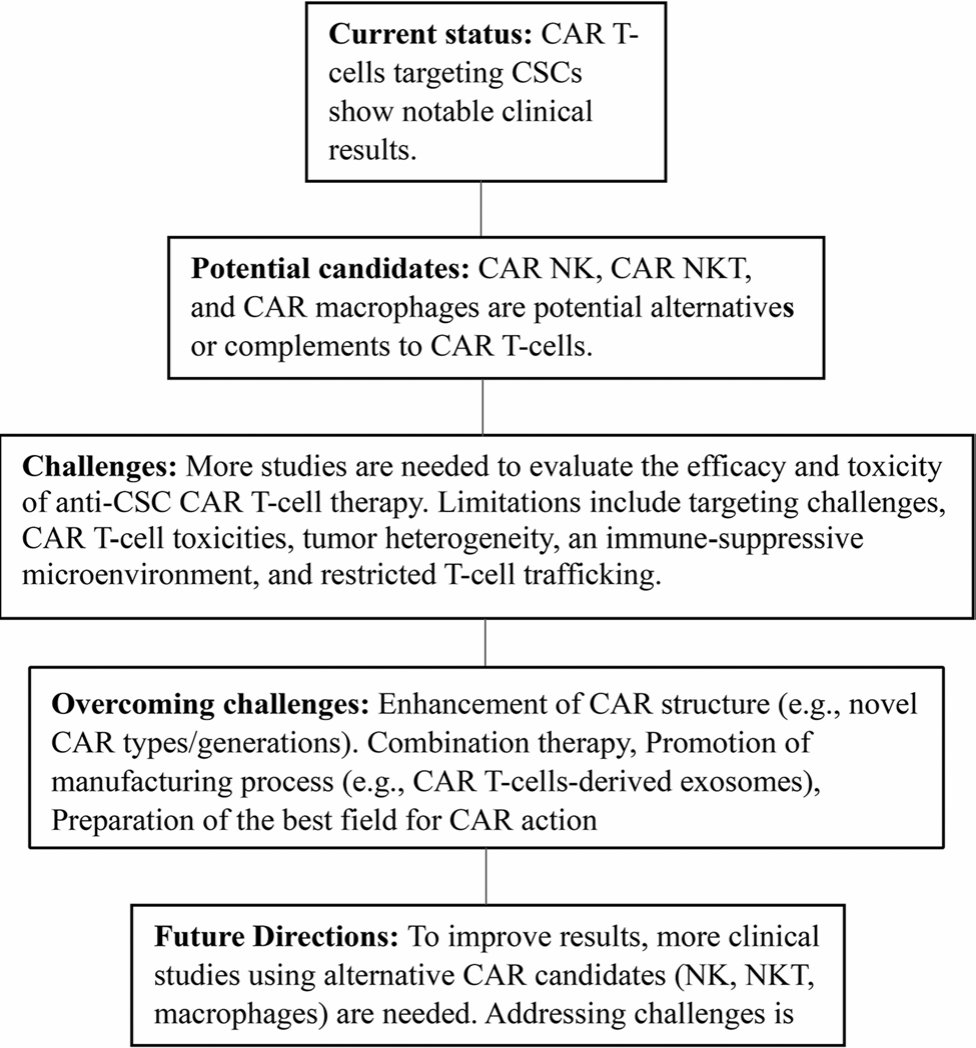

## Data Availability

No datasets were generated or analysed during the current study.

## Abbreviations

ACT: Adoptive cell therapy
CAR: Chimeric antigen receptor
TAA: Tumor associated antigen
CSC: Cancer stem cell
FDA: Food and drug administration
NK: Natural killer
NKT: Natural killer T
ScFV: Single-chain variable fragment
M: Macrophage
MDSC: Myeloid-derived suppressor cell
DC: Dendritic cell
T-reg: T-regulatory cell
ADCC: Antibody-dependent cellular toxicity
GM-CSF: Granulocyte-macrophage colony-stimulating factor
ROS: Reactive oxygen species
MMP: Metalloproteinase
ECM: Extracellular matrix
ALDH: Aldehyde dehydrogenases
CRS: Cytokine release syndrome
GvHD: Graft-versus-host disease
VEGF-A: Vascular endothelial growth factor A
TAM: Tumor-associated macrophage
DC: Dendritic cell
MHC: Major histocompatibility complex
AML: Acute myeloid leukemia
CML: Chronic myeloid leukemia
MM: Multiple myeloma
ALL: Acute lymphoblastic leukemia
CLL: Chronic lymphoblastic leukemia
LIC: Leukemic-initiating cell
CLL-1: C-type lectin-like molecule 1
FLT3: FMS-like tyrosine kinase 3
IL-1RAP: Interleukin-1 receptor accessory protein
CIK: Cytokine-induced killer
BsAbs: Bispecific antibodies
iPSC: Induced pluripotent stem cell
NKG2DL: Natural killer group 2 member D ligand
CRISPR/Cas9: Clustered regularly interspaced short palindromic repeats and CRISPR-associated protein 9
ATRA: All-trans retinoic acid
TNF-α: Tumor necrosis factor alpha
IL: Interleukin
HAM: Hypomethylating agents
LAM: Leukemia-associated macrophage
LSC: Leukemia stem cell
CR: Complete remission
PR: Partial remission
ICANS: Immune effector cell-associated neurotoxicity syndrome
MRD: Minimal residual disease
MLFS: Morphological leukemia-free state
SD: Stable disease
ORR: Objective response rate
CRi: Complete remission with incomplete hematological recovery
Flu: Fludarabine
Cy: Cyclophosphamide
Ara-C: Cytarabine
MTZ: Mitoxantrone
CRC: Colorectal cancer
EMD: Extramedullary disease
PFS: Progression-free survival
OS: Overall survival
EASIX: Endothelial activation and stress index
SCT: Allogenic stem cell transplantation
GCS: Glioblastoma stem cell
SoC: Standard of care
TGF-β: Transforming growth factor beta
TME: Tumor microenvironment
EGFR: Epidermal growth factor
CTL: Cytotoxic T lymphocyte
PSCA: Prostate stem cell antigen
PCa: Prostate cancer
FIR: Fractionated irradiation
CRPC: Castrate-resistant prostate cancer
mcDNA: Minicircle DNA
PSMA: Prostate-specific membrane antigen
BCSC: Breast cancer stem cell
TF: Tissue factor
ICON: Antibody-like immunoconjugate
PD-L1: Programmed cell death ligand 1
IFN: Interferon
SCLC: Small-cell lung cancer
NSCLC: Non-small-cell lung cancer
csGRP78: Cell surface glucose-regulated protein 78
HCC: Hepatocellular carcinoma
CCA: Cholangiocarcinoma
BBB: Blood-brain barrier
LDH: Lactate dehydrogenase
ICAM-1: Intercellular adhesion molecule-1
VCAM-1: Vascular cell adhesion protein-1
TAN: Tumor-associated neutrophiles
BiTE: Bispecific T-cell engager
FAP: Fibroblast activation protein
ICI: Immune checkpoint inhibitor
JAK: Janus kinase
STAT: Signal Transducer and Activator of Transcription
CAF: Cancer associated fibroblast
SNIP: Signal Neutralization, by inhibitable proteinase
TAPi: Transporter associated with antigen processing
CIITA: Class II MHC transactivator
HIT: HLA-independent T cell receptor
TRuC: TCR fusion construct
EV: Peptide construct
PGC-1α: PPAR-gamma coactivator 1 alpha
sgRNA: Single guide RNA
DSB: Double-strand break
crRNA: CRISPR RNA
tracrRNA: Trans-activating CRISPR RNA
TRBC: T-cell receptor beta constant
TRAC: T-cell receptor alpha constant
TSCM: Stem cell memory T-cell
MPEI: Mannose-conjugated polyethyleneimine
AI: Artificial intelligence
AUC: Areas under the curves
